# Federated inference and belief sharing

**DOI:** 10.1016/j.neubiorev.2023.105500

**Published:** 2024-01

**Authors:** Karl J. Friston, Thomas Parr, Conor Heins, Axel Constant, Daniel Friedman, Takuya Isomura, Chris Fields, Tim Verbelen, Maxwell Ramstead, John Clippinger, Christopher D. Frith

**Affiliations:** a Wellcome Trust Centre for Neuroimaging, Institute of Neurology, University College London, UK; b VERSES AI Research Lab, Los Angeles, CA 90016, USA; c Department of Collective Behaviour, Max Planck Institute of Animal Behavior, 78457 Konstanz, Germany; d Centre for the Advanced Study of Collective Behaviour, 78457 Konstanz, Germany; e Department of Biology, University of Konstanz, 78457 Konstanz, Germany; f School of Engineering and Informatics, The University of Sussex, Brighton, UK; g Department of Entomology and Nematology, University of California, Davis, Davis, CA, USA; h Active Inference Institute, Davis, CA 95616, USA; i Brain Intelligence Theory Unit, RIKEN Center for Brain Science, Wako, Saitama 351-0198, Japan; j Allen Discovery Center at Tufts University, Medford, MA 02155, USA; k Bioform Labs, and MIT Media Lab, Boston, USA; l Institute of Philosophy, School of Advanced Studies, University of London, UK

**Keywords:** Active inference, Distributed cognition, Federated learning, Structure learning, Message passing

## Abstract

This paper concerns the distributed intelligence or federated inference that emerges under belief-sharing among agents who share a common world—and world model. Imagine, for example, several animals keeping a lookout for predators. Their collective surveillance rests upon being able to communicate their beliefs—about what they see—among themselves. But, how is this possible? Here, we show how all the necessary components arise from minimising free energy. We use numerical studies to simulate the generation, acquisition and emergence of language in synthetic agents. Specifically, we consider inference, learning and selection as minimising the variational free energy of posterior (i.e., Bayesian) beliefs about the states, parameters and structure of generative models, respectively. The common theme—that attends these optimisation processes—is the selection of actions that minimise expected free energy, leading to active inference, learning and model selection (a.k.a., structure learning). We first illustrate the role of communication in resolving uncertainty about the latent states of a partially observed world, on which agents have complementary perspectives. We then consider the acquisition of the requisite language—entailed by a likelihood mapping from an agent's beliefs to their overt expression (e.g., speech)—showing that language can be transmitted across generations by active learning. Finally, we show that language is an emergent property of free energy minimisation, when agents operate within the same econiche. We conclude with a discussion of various perspectives on these phenomena; ranging from cultural niche construction, through federated learning, to the emergence of complexity in ensembles of self-organising systems.

## Highlights

• Communication—and language in particular—is an emergent property of agents that seek evidence for generative models of their shared world.
• Nested free energy minimising—evidence maximising—processes explain the emergence of language and its transmission over generations.
• Reading these processes as inference integrates perspectives on communication; from generalised synchrony to cultural niche construction.

## 1 Introduction

This paper concerns the genesis of communication and distributed cognition in ensembles of agents who share the same world—and internal or generative model of that world (Bahrami et al., 2010, Constant et al., 2019, Friston and Frith, 2015, Friston et al., 2022b, Frith and Wentzer, 2013, Kairouz et al., 2021, Levin, 2019, Vasil et al., 2020). It uses simulations of agents who broadcast their beliefs about inferred states of the world to other agents, enabling them to engage in joint inference and learning. Consider, for example, three birds monitoring their environment for predators. Each bird will have more or less precise beliefs about the location and disposition of potential predators. If they broadcast these beliefs to each other, a shared belief about the world could emerge that would be more precise than any individual’s belief. We simulate the requisite belief-sharing using a series of free energy minimising processes to reproduce active inference, learning and selection, where selection refers to the selection of structures or functional forms for generative models of the sensed world.

By equipping agents with a shared generative model—of a shared world—one can effectively distribute or federate Bayesian belief updating over multiple agents—where this federation rests upon the sharing of posterior beliefs about hidden (a.k.a., latent) states of affairs (Kairouz et al., 2021). Clearly, for this to work, collocutors must share a common ground or frame of reference (Adank et al., 2010, Allan, 2013, Barsalou, 2008, Chomsky, 2006, Garrod and Pickering, 2009). In other words, there has to be an isomorphism between the beliefs of one agent and those of another. This necessitates generative models in which some belief structures, or representations, are conserved over agents. Beliefs over this common ground can, in principle, be shared among agents—so that every agent inherits and assimilates the perspective of others. Clearly, to realise this kind of belief-sharing, it is necessary to generate observable outcomes or messages that can be recognised by other agents. In turn, this necessitates a common mapping between shared beliefs and their overt expression (e.g., language); namely, a shared likelihood model responsible for language generation and recognition. In what follows, we will focus on the *deployment*, *acquisition* and *emergence* of this likelihood model, foregrounding the role of *active inference*, *active learning* and *active selection*, respectively. This emergence recapitulates numerical studies of collective intelligence; in which the alignment between agents’ free energy minima “emerges endogenously from the dynamics of interacting AIF [active inference] agents themselves” (Kaufmann et al., 2021).

There are many perspectives that one could take on these nested processes; ranging from peer-to-peer message passing and belief-sharing in computer science to the emergence of language in developmental and evolutionary psychology (Constant et al., 2018, Frith, 2010, Ghazanfar and Takahashi, 2014, Hauser et al., 2002, Heyes, 2018, Kastel and Hesp, 2021, Laland et al., 2016, Steels, 2011, Tomasello, 2016, Veissiere et al., 2019). We will pick up these perspectives in the discussion but first rehearse their theoretical foundations through the lens of the free energy principle (Friston et al., 2022a, Ramstead et al., 2022).

The simulations used in this study are realisations of free energy minimising processes at distinct temporal scales that can be interpreted in terms of inference, learning and selection. Free energy minimisation is just a way of describing self organisation in open—and therefore nonequilibrium—systems that are coupled to each other. Here, we consider several agents that are in exchange with a common environment, and each other. Each agent can be regarded as a (Bayesian) belief updating process, where each agent entails a generative model of her environment.^1^ The beliefs in question pertain to latent states, parameters and structures: all of which change to minimise a free energy functional; namely, a variational free energy that incorporates expected free energy (Parr and Friston, 2019). Expected free energy could be regarded as definitive of active inference, in the sense that it scores the likelihood of various actions, while variational free energy scores the marginal likelihood of observations, under some (Bayesian) beliefs about how observations were caused.

In the current application of the free energy principle, we generalise the notion of action to anything that has consequences. At the level of inference, action corresponds to sampling or selecting things that are expected to minimise free energy (Friston et al., 2011). At the level of learning, action involves updating beliefs about the parameters of the generative model: e.g., active learning (Mackay, 1992, Schmidhuber, 2010, Vigorito and Barto, 2010). Similarly, in model selection or structure learning (Gershman and Niv, 2010, Pellet and Elisseeff, 2008, Smith et al., 2020, Tervo et al., 2016) action ‘selects’ certain priors over parameters. We will see later, that—from the point of view of model parameters and structure—selecting updates that minimise expected free energy is equivalent to maximising the mutual information of the likelihood mapping between observable consequences and latent or unobservable causes, under certain constraints.

Minimising variational free energy—with respect to beliefs over states and parameters—is equivalent to maximising the model evidence (a.k.a., marginal likelihood) of observations, under the agent's generative model (Winn and Bishop, 2005). This is sometimes referred to as self-evidencing (Fields et al., 2021a, Hohwy, 2016). Put simply, to self-evidence means to get a better grip on the world—a grip that may be tighter when agents pull together by sharing their beliefs (Bruineberg et al., 2018, Constant et al., 2019). Interesting, this rests on getting a grip on the common ground that enables agents to make sense of each other.

In what follows, we unpack the emergence of self-evidencing and common ground in three steps. We first demonstrate the role of belief-sharing, in resolving uncertainty about hidden states, when their observable consequences can only be seen by one agent at a time. We then illustrate the acquisition of language (i.e., likelihood mappings) using active learning or accumulation of Dirichlet counts, where a child learns from its parents and conspecifics. Finally, we illustrate the emergence of language (i.e., precise and shared likelihood mappings) in an ensemble of agents. This emergence rests upon structure learning, in which priors over the Dirichlet counts of likelihood tensors are updated to minimise expected free energy. The ensuing active (Bayesian) model selection uses Bayesian model reduction (Friston et al., 2018).

This paper comprises four key sections. The first provides a summary of active inference under generative models of discrete state spaces; in other words, where the hidden or latent states, causing observations, are in one discrete state or another. This section offers a technical preamble for readers who want to understand the mechanics of belief updating used in subsequent sections (and other applications of active inference to discrete models). The second section focuses on the special issue of how beliefs are shared among agents, where each agent is equipped with her own generative model of the world. We will use a simple setup to illustrate belief sharing, in which three agents broadcast their beliefs about the causes of their sensations. All three agents share a generative model of what they would ‘see’—from their unique perspectives—given states of affairs in their shared world. Crucially, they also share a generative model of what they would ‘hear’ if beliefs about those states where articulated or broadcast. The implicit communication enables the updating of beliefs based upon what all three agents can see; effectively, accumulating evidence through ‘many pairs of eyes’. The augmented efficiency of belief updating—and its neuronal correlates—rest upon assuming a shared generative model, in which the mapping between each agent’s beliefs and what she says (and hears) is conserved over agents. In the second section, we ask whether this shared mapping could be learned by a naïve agent (e.g., an infant) exposed to the same visual and auditory scenes as the communicating agents (e.g., parents). We will see that the requisite mapping emerges as a consequence of minimising free energy, rendering the sensorium as predictable as possible. These simulations assume the existence of a language that can be learned. In the final simulations, we ask whether free energy minimising processes are sufficient to account for the de novo emergence of likelihood mappings—that underwrite linguistic exchange—by exposing three naïve agents to shared visual scenes. Again, we will see the emergence of precise likelihood mappings that are conserved over agents. In short, communication appears to be an emergent property of agents who broadcast their beliefs about a shared world.

In summary, we hoped to show—using numerical analyses—that federated inference and learning emerges from minimising (variational, expected and reduced) free energy. Formally, this tuple of free energy minimising processes provides a first principles account of (state) inference, (parameter) learning and (model) selection that can be applied to any generative model and implicit agent. Our focus here is on its application to an ensemble of agents to evince self-evidencing through belief-sharing. This could be read as a move towards a formal account of consciousness in the pre-Cartesian sense of sharable knowledge (i.e., *con* – ‘together’ and *scire* – ‘to know’). More practically, it might speak to the design principles for ecosystems of intelligent agents (Friston and Frith, 2015, Friston et al., 2022b, Frith and Wentzer, 2013).^2^

## 2 Active inference and free energy

Active inference rests upon a *generative model* of observable outcomes. This model is used to infer the most likely causes of outcomes in terms of expected states of the world. These states (and paths) are latent or *hidden* because they can only be inferred through observations. Some paths are considered controllable in the sense they can be changed by acting. Crucially, certain observations depend upon action (e.g., where one is looking), which requires the generative model to entertain expectations about outcomes under different combinations of actions (i.e., policies).^3^ These expectations are optimised by minimising *variational free energy*. Crucially, the prior probability of a policy depends upon its *expected free energy*. Expected free energy has a number of familiar special cases; including, expected utility, intrinsic value, Bayesian surprise, mutual information, *etc*. Having evaluated the expected free energy of each policy—and implicitly their prior likelihood—the most likely action can be selected. This action generates a new outcome and the (perception-action) cycle starts again (Parr et al., 2022).

### 2.1 The generative model

**Fig. 1:**
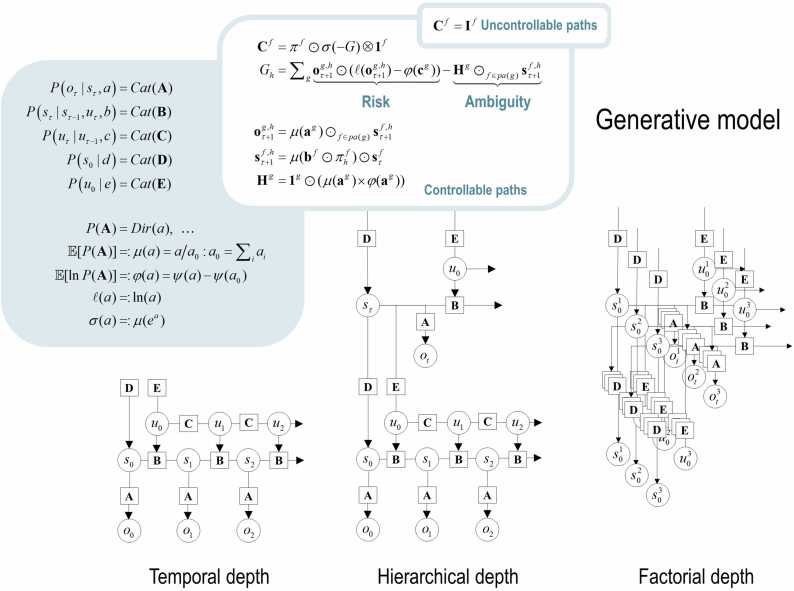
Generative models as agents. A generative model specifies the joint probability of outcomes or consequences and their causes; namely, hidden states. This joint distribution can be expressed in terms of a *likelihood* (the probability of consequences given their causes) and *priors* (over causes). When a prior depends upon a random variable it is called an *empirical prior*. Here, the likelihood is specified by a tensor A, encoding the probability of an outcome under every combination of *states* (*s*). The empirical priors in this instance pertain to transitions among hidden states B that depend upon *paths* (*u*), whose transition probabilities are encoded in C. The key aspect of this generative model is that certain (controllable) paths are more probable a priori if they minimise their expected free energy (G), expressed in terms of *risk* and *ambiguity* (lower white panel). If the path is not controllable, it remains unchanged during the epoch in question (upper white panel), where E specifies the initial probability of each path. The left panel provides the functional form of the generative model in terms of categorical (*Cat*) distributions. The lower equalities list the various operators required for the variational message-passing—and updating Bayesian beliefs about hidden states and paths—detailed in Fig. 2. These functions are taken to operate on each column of their tensor arguments. The graph on the lower left depicts the generative model as a probabilistic graphical model that foregrounds the implicit *temporal depth* implied by priors over state transitions and paths. This example only shows dependencies for uncontrollable paths. When equipped with *hierarchical depth* the POMDP acquires a separation of temporal scales. This follows from a construction in which the succession of states at a higher level generates the initial states (via the D tensor) and paths (via the E tensor) at the lower level. This means higher levels unfold more slowly than lower levels, thereby furnishing empirical priors (c.f., inductive biases) that contextualise the dynamics of lower level states. At each hierarchical level, the hidden states and accompanying paths are factored to endow the model with *factorial depth*. In other words, the model or agent ‘carves nature at its joints’ into factors that conspire or interact to generate outcomes (or the initial states and paths at lower levels). This means context-sensitive contingencies are mediated by the tensors mapping from one level to the next (D and E) or outcomes (A). The subscripts in this figure pertain to time, while the superscripts denote different factors (*f*), outcome modalities (*g*) and combinations of paths over factors (*h*). Tensors and matrices are denoted by uppercase bold, while posterior expectations are in lowercase bold. The matrix *π* encodes the probability over paths, under each policy (where *π*_*h*_ denotes the probability over paths for policy *h*). The ⊙ notation implies a generalised inner (i.e., dot) product or tensor contraction, while × denotes the Hadamard (element by element) product. *ch*(.) and *pa*(.) return the children and parents of any node; namely, the co-domain and domain, respectively. Finally, *ψ* denotes the digamma function (the logarithmic derivative of the gamma function).

Fig. 1 provides a schematic specification of the generative model used for the sorts of problems considered in this paper. In brief, outcomes at any particular time depend upon hidden *states*, while transitions among hidden states depend upon *paths*. Note that paths are random variables in the sense that a particle can have both a position (i.e., a state) and momentum (i.e., a path). Paths may or may not depend upon action. The resulting partially observed Markov decision process (POMDP) is specified by a set of tensors. The first set **A**, maps from hidden states to outcome modalities; for example, exteroceptive (e.g., visual) or proprioceptive (e.g., eye position) modalities. These parameters encode the likelihood of an outcome given their hidden causes. The second set **B** prescribes transitions among the *factors* of hidden states, under a particular path. These factors correspond to different states of the world, like the location or nature of an object. The remaining tensors encode prior beliefs about paths **C**, and initial states **D**. The tensors—encoding probabilistic mappings or contingencies—are generally parameterised as Dirichlet distributions, whose sufficient statistics are concentration parameters or *Dirichlet counts*. These count the number of times a particular combination of states or outcomes has been observed. We will focus on learning the likelihood model, encoded by Dirichlet counts, ***a***.

The generative model in Fig. 1 means that outcomes are generated as follows: first, a policy is selected using a softmax function of expected free energy. Sequences of hidden states are generated using the probability transitions specified by the selected combination of paths (i.e., policy). Finally, these hidden states generate outcomes in one or more modalities. Perception or inference about hidden states (i.e., state estimation) corresponds to inverting a generative model, given a sequence of outcomes, while learning corresponds to updating model parameters. Perception therefore corresponds to accumulating evidence for beliefs about hidden states and paths, while learning corresponds to accumulating knowledge in the form of Dirichlet counts. The requisite expectations constitute the sufficient statistics (s,u,a) of posterior beliefs $Q(s,u,a)=Q_{s}(s)Q_{u}(u)Q_{a}(a)$. Because we are dealing with discrete states, (s,u,a) are just the expected probability of states, paths and likelihood parameters, respectively. The implicit factorisation of this approximate posterior effectively partitions model inversion into inference, planning and learning.

**Fig. 2:**
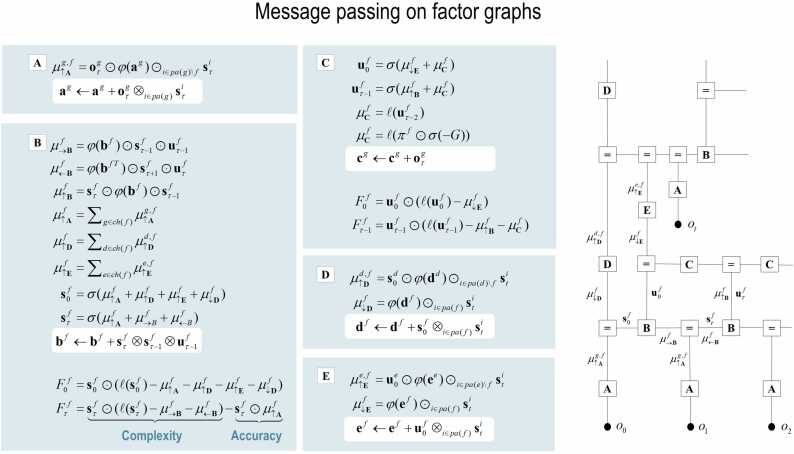
Belief updating and variational message passing: the right panel presents the generative model as a factor graph, where the nodes (square boxes) correspond to the factors of the generative model (labelled with the associated tensors). The edges connect factors that share dependencies on random variables. The leaves of (filled circles) correspond to known variables, such as observations (*o*). This representation is useful because it scaffolds the message passing—over the edges of the factor graph—that underwrite inference and planning. The functional forms of these messages are shown in the left-hand panels. For example, the expected path—in the first equality of panel C—is a softmax function of two messages. The first is a descending message $\mu_{↓E}^{f}$ from E that inherits from expectations about hidden states at the hierarchical level above. The second is the log-likelihood of the path based upon expected free energy. This message depends upon Dirichlet counts scoring preferred outcomes—in modality *g*—encoded in $c^{g}$ (see Fig. 1). The two expressions for $\mu_{C}^{f}$ correspond to uncontrolled and controlled paths, respectively. Note that the posterior expectation of the initial path is learned, in a context sensitive fashion, by accumulation in $e^{f}$, which describes how policies can become habits. The updates in the lighter panels correspond to learning; i.e., updating Bayesian beliefs about parameters (adopting the Einstein summation with respect to *τ*). Similar functional forms for other messages can be derived, by direct calculation. These furnish the fixed points (i.e., free energy minimisers), which render this kind of variational message passing a fixed-point iteration scheme. The requisite functions are defined in Fig. 1. The ⊙ notation implies a generalised inner product or tensor contraction, while ⊗ denotes an outer product. *ch*(·) and *pa*(·) return the children and parents of any node; namely, the domain and co-domain, respectively. There is a link (i.e., edge) between a parent and child, if the co-domain of the parent factor (i.e., node) constitutes a domain of a child. This means that each edge—and (the co-domain of) each factor—is uniquely associated with a state, path or outcome.

### 2.2 Variational free energy and inference

In variational Bayesian inference (a.k.a., approximate Bayesian inference), model inversion entails the minimisation of variational free energy with respect to the sufficient statistics of approximate posterior beliefs. This can be expressed as follows, where—for clarity—we will deal with a single factor, such that the policy (i.e., combination of paths) becomes the path, π=uand omit dependencies on previous states:

(1) $$\begin{array}{c} Q\left(s_{\tau },u_{\tau },a\right)=\text{↓arg }\min_{Q}F\\ F=E_{Q}\left[\;\ln\;\underset{\mathrm{posterior}}{\underset{⏟}{Q\left(s_{\tau },u_{\tau },a\right)}}-\;\ln\;\underset{\mathrm{likelihood}}{\underset{⏟}{P\left(o_{\tau }\right|s_{\tau },u_{\tau },a)}}-\;\ln\;\underset{\mathrm{prior}}{\underset{⏟}{P\left(s_{\tau },u_{\tau },a\right)}}\right]\\ =\underset{\mathrm{divergence}}{\underset{⏟}{D_{KL}\left[Q(s_{\tau },u_{\tau },a)||P(s_{\tau },u_{\tau },a\left|o_{\tau }\right)\right]}}-\;\ln\;\underset{\mathrm{evidence}}{\underset{⏟}{P\left(o_{\tau }\right)}}\\ =\underset{\mathrm{complexity}}{\underset{⏟}{D_{KL}\left[Q\left(s_{\tau },u_{\tau },a\right)||P\left(s_{\tau },u_{\tau },a\right)\right]}}-\underset{\mathrm{accuracy}}{\underset{⏟}{E_{Q}\left[\;\ln\;P\left(o_{\tau }\right|s_{\tau },u_{\tau },a)\right]}} \end{array}$$

This equation specifies the (approximate posterior) beliefs $Q(s_{\tau },u_{\tau },a)$—about states, paths and likelihoods—as those beliefs that minimise variational free energy, where variational free energy has been expressed in three equivalent functional forms; each affording a complementary interpretation. Here, $P(o_{\tau },s_{\tau },u_{\tau },a)$is the generative model; namely, the probability distribution over causes, $\left(s_{\tau },u_{\tau },a\right)$ and observable consequences, $\left(o_{\tau }\right)$ at time τ.

Because the (KL) divergences cannot be less than zero, the penultimate equality means that free energy is zero when the (approximate) posterior is the true posterior. At this point, the free energy becomes the negative log evidence for the generative model (Beal, 2003). This means minimising free energy is equivalent to maximising model evidence, which is equivalent to minimising the complexity of accurate explanations for observed outcomes.

Minimising free energy therefore ensures expectations encode posterior beliefs, given observed outcomes. This is inference. Planning emerges under active inference by placing priors over (combinations of) paths to minimise expected free energy (Friston et al., 2015):

(2) $$\begin{array}{c} G\left(u\right)=E_{Q_{u}}[\;\ln\;Q(s_{\tau +1},a\left|u\right)-\;\ln\;Q(s_{\tau +1},a|o_{\tau +1},u)-\;\ln\;P(o_{\tau +1}\left|c\right)]\\ =-\underset{\mathrm{expectedinformationgain}\left(\mathrm{learning}\right)}{\underset{⏟}{E_{Q_{u}}[\;\ln\;Q\left(a\right|s_{\tau +1},o_{\tau +1},u)-\;\ln\;Q(a\left|u\right)]}}-\\ \underset{\mathrm{expectedinformationgain}\left(\mathrm{inference}\right)}{\underset{⏟}{E_{Q_{u}}[\;\ln\;Q\left(s_{\tau +1}\right|o_{\tau +1},u)-\;\ln\;Q(s_{\tau +1}\left|u\right)]}}\underset{\mathrm{expectedcost}}{\underset{⏟}{-E_{Q_{u}}[\;\ln\;P(o_{\tau +1}\left|c\right)]}}\\ =-\underset{\mathrm{novelty}}{\underset{⏟}{E_{Q_{u}}[D_{KL}[Q\left(a\right|s_{\tau +1},o_{\tau +1},u)||Q(a\left|u\right)]]}}+\\ \underset{\mathrm{risk}}{\underset{⏟}{D_{KL}[Q(o_{\tau +1}\left|u\right)||P(o_{\tau +1}\left|c\right)]}}\underset{\mathrm{ambiguity}}{\underset{⏟}{-E_{Q_{u}}[\;\ln\;Q\left(o_{\tau +1}\right|s_{\tau +1},u)]}} \end{array}$$

Here, $Q_{u}=Q(o_{\tau +1},s_{\tau +1},a|u)=P(o_{\tau +1},s_{\tau +1},a|u,o_{0},\ldots ,o_{\tau })=P(o_{\tau +1}|s_{\tau +1},a)Q(s_{\tau +1},a|u)$ is the posterior predictive distribution over parameters, hidden states and outcomes at the next time step, under a particular path. Note that the expectation is over *observations in the future* that become random variables; hence, *expected* free energy. This means that preferred outcomes—that subtend expected cost and risk—are prior beliefs, which constrain the implicit planning as inference (Attias, 2003, Botvinick and Toussaint, 2012, Van Dijk and Polani, 2013).

One can also express the prior over the parameters in terms of an expected free energy, where, marginalising over paths:

(3) $$\begin{array}{c} P\left(a\right)=\sigma \left(-G\right)\\ G\left(a\right)=E_{Q_{a}}\left[\;\ln\;P(s\left|a\right)-\;\ln\;P\left(s\right|o,a)-\;\ln\;P(o\left|c\right)\right]\\ =\underset{\mathrm{expected}\;\;\mathrm{information}\;\;\mathrm{gain}}{\underset{⏟}{E_{Q_{a}}\left[\;\ln\;P\left(s\left|a\right)-\;\ln\;P\left(s\right|o,a\right)\right]}}\underset{\mathrm{expected}\;\;\mathrm{cost}}{\underset{⏟}{-E_{Q_{a}}\left[\;\ln\;P(o\left|c\right)\right]}}\\ =-\underset{\mathrm{mutual}\;\;\mathrm{information}}{\underset{⏟}{E_{Q_{a}}[D_{KL}[P(o,s\left|a\right)||P(o\left|a\right)P(s\left|a\right)]}}\underset{\mathrm{expected}\;\;\mathrm{cost}}{\underset{⏟}{-E_{Q_{a}}\left[\;\ln\;P(o\left|c\right)\right]}} \end{array}$$

where $Q_{a}=P(o|s,a)P(s|a)=P(o,s|a)$ is the joint distribution over outcomes and hidden states, encoded by the Dirichlet parameters, *a*. Note that the Dirichlet parameters encode the mutual information, in the sense that they implicitly encode the joint distribution over outcomes and their hidden causes. When normalising each column of the *a* tensor, we recover the likelihood distribution (as in Fig. 1); however, we could normalise over every element, to recover a joint distribution [we will use this later in Eq. (9)].

Expected free energy can be regarded as a universal objective function that augments mutual information with expected costs or constraints. Constraints—parameterised by *c*—reflect the fact that we are dealing with open, nonequilibrium, systems with characteristic outcomes, *o*. This can be read as an expression of the constrained maximum entropy principle that is dual to the free energy principle (Ramstead et al., 2022). Alternatively, it can be read as a constrained principle of maximum mutual information or minimum redundancy (Ay et al., 2008, Barlow, 1961, Linsker, 1990a, Olshausen and Field, 1996b). In machine learning, this kind of objective function underwrites disentanglement (Higgins et al., 2021, Sanchez et al., 2019), and generally leads to sparse representations (Gros, 2009, Olshausen and Field, 1996b, Sakthivadivel, 2022b, Tipping, 2001).

When comparing the expressions for expected free energy in (2) with variational free energy in (1), the expected divergence becomes expected information gain. Expected information gain about the parameters and states are sometimes associated with distinct epistemic affordances; namely, novelty and salience, respectively (Schwartenbeck et al., 2019). Similarly, expected log evidence becomes expected value, where value is the logarithm of prior preferences. The last equality provides a complementary interpretation; in which the expected complexity becomes risk, while expected inaccuracy becomes ambiguity.

There are many special cases of minimising expected free energy. For example, maximising expected information gain maximises (expected) Bayesian surprise (Itti and Baldi, 2009), in accord with the principles of optimal experimental design (Lindley, 1956). This can also be interpreted in terms of the principle of maximum mutual information or minimum redundancy (Barlow, 1961, Laughlin, 2001, Linsker, 1990b, Olshausen and Field, 1996a). This resolution of uncertainty is closely related to artificial curiosity (Schmidhuber, 1991, Still and Precup, 2012) and speaks to the value of information (Howard, 1966), particularly in the context of evincing information necessary to realise preferred outcomes. See (Meder and Nelson, 2012, Nelson et al., 2010), who compare different models of information gain in perceptual decision-making.

Expected complexity or risk is the same quantity minimised in risk sensitive or KL control (Klyubin et al., 2005, van den Broek et al., 2010), and underpins (free energy) formulations of bounded rationality based on complexity costs (Braun et al., 2011, Ortega and Braun, 2013) and related schemes in machine learning; e.g., Bayesian reinforcement learning (Ghavamzadeh et al., 2016). More generally, minimising expected cost subsumes Bayesian decision theory (Berger, 2011).

### 2.3 Belief updating

In variational treatments, the sufficient statistics encoding posterior expectations are updated by minimising variational free energy. Fig. 2 illustrates these updates in the form of variational message passing (Dauwels, 2007, Friston et al., 2017b, Winn and Bishop, 2005). Although the updates look complicated, they lend themselves to neurobiological implementation—at a certain level of analysis—in a straightforward fashion (Friston et al., 2016, Friston et al., 2014). This is because the updates only require nonlinear mappings and sum-product (tensor) operations.

For example, expectations about hidden states are a softmax function of messages that are linear combinations of other expectations and observations.

(4) $$\begin{array}{c} s_{\tau }^{f}=\sigma (\mu_{↑A}^{f}+\mu_{\to B}^{f}+\mu_{\leftarrow B}^{f})\\ \mu_{↑A}^{f}=\sum_{g\in ch\left(f\right)}\mu_{↑A}^{g,f}\\ \mu_{↑A}^{g,f}=o_{\tau }^{g}⊙\varphi \left(a^{g}\right){⊙}_{i\in pa\left(g\right)\backslash f}s_{\tau }^{i} \end{array}$$

In this example, conditional expectations about hidden states (of factor *f*) are a normalised exponential of several messages that can be read as log probabilities. Here, these messages correspond to a log likelihood due to observations and log priors from the past and future beliefs about hidden states (that entail dynamics). In turn, the log likelihood message is itself a mixture of ascending messages from those outcome modalities that are the children of the factor in question. The final equality means that each ascending messages is a linear mixture^4^ of expected states and observations, weighted by (digamma) functions of the Dirichlet counts, which correspond to the parameters of the likelihood model (c.f., connections weights). In practice, one can use an upper bound for the likelihood messages. From Jensen's inequality (assuming a single parent for clarity) 5:

(5) $$\begin{array}{c} \mu_{↑A}^{g}=o_{\tau }^{g}⊙\varphi \left(a^{g}\right)\\ =E_{Q(o,a)}[\;\ln\;P\left(A^{g}\right)]\leq \;\ln\;E_{Q(o,a)}[P\left(A^{g}\right)]\\ =\ell (o_{\tau }^{g}⊙\mu \left(a^{g}\right)) \end{array}$$

This functional form can exploit the sparsity of likelihood tensors to finesse the von Neumann bottleneck. From a biological perspective, this just means that there is no contribution to a message if the connection is absent or has been removed via structure learning. In fact, these are the messages that are used in belief propagation, during online belief updating (that eschews backward message passing or variational approximations) (Kschischang et al., 2001, Parr et al., 2019).

## 3 Active inference and belief sharing

The setup used to illustrate belief-sharing considers three agents that can be thought of as birds (i.e., sentinels) maintaining surveillance for a predator—or sisters hiding from their mother in the garden. Crucially, each agent has a restricted field of view, covering about a third of the horizon. In addition to visual and proprioceptive (gaze-related) observation modalities, each agent can also hear the others (but not herself). To realise belief-sharing, agents have a simple (identity) likelihood mapping between posterior beliefs—about latent states that are shareable among agents—and output modalities or communication channels (e.g., talking). In subsequent sections we will consider the emergence of the requisite likelihood mappings. In this section, we will focus on the mechanisms and benefits of communication afforded by belief sharing among three agents, who have complementary views of the world.

**Fig. 3:**
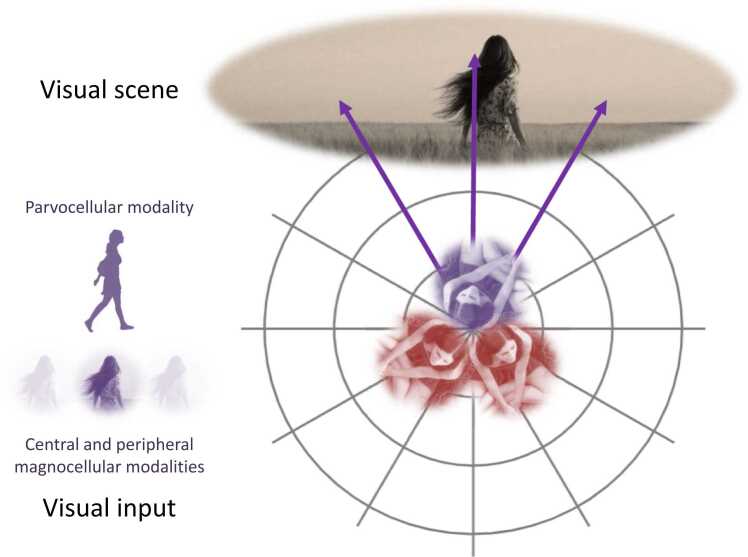
The agents’ worldview. This graphic illustrates how the world generates (visual) observations for our agents. In this setup, there are three agents, sitting back-to-back, who survey a scene in which a subject (e.g., predator or person) is nearby. The subject can be standing *still* or encircling the agents in a clockwise (*right*) or anticlockwise (*left*) direction; either *close* or *near* to the agents. When the subject is *close*, agents can discern whether it is a *friend* or a *foe* (e.g., conspecific or a predator). The requisite visual observations have four modalities: a central foveal input—we associate with the parvocellular stream—that classifies the subject as either a proximate *friend* or *foe* or *someone* in the distance (c.f., the product of an image classification algorithm). The remaining three (magnocellular) modalities are low spatial frequency reports of contrast energy, in central and peripheral regions of the visual field (c.f., LIDAR detectors). Each contrast energy has three levels; *close*, *near* or *none*). Each agent can only see what is in front of her but can look to the *right* or *left*—to extend her field of view (purple arrows). In the example of visual input above, the purple agent is looking straight ahead at someone, and her foveal (parvocellular) observation is someone in the distance. The remaining visual modalities (lower circles) register *near* contrast energy in the centre of the visual field with *none* on the right and left. The latent states of the environment generating these observations are described in the subsequent figure.

**Fig. 4:**
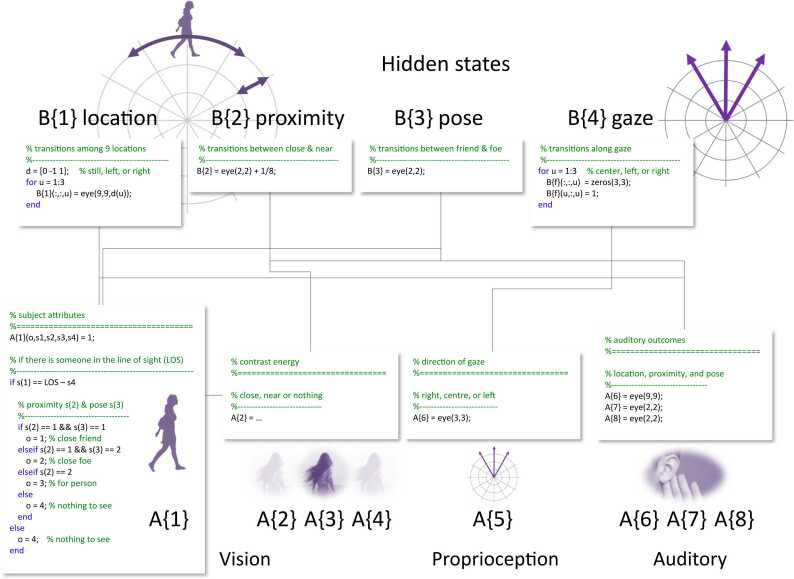
states of affairs in the world. This figure summarises the hidden states generating outcomes in terms of factors and their accompanying levels. It is presented in a way that will be familiar to people specifying generative models for active inference; specifically, using (Matlab) pseudocode to illustrate how one specifies likelihood mappings and transition priors in terms of simple matrices and logical expressions. In this generative model, there are four factors corresponding to the allocentric position (*location*) of the subject, whether the subject is close or near (*proximity*), whether the subject is friendly or not (*pose*) and where the agent is currently looking (*gaze*). The transitions among the states of these four factors are encoded by prior B tensors. These tensors are probability transition matrices with multiple slices; where each slice corresponds to a particular path, trajectory or action. Only the location and gaze factors have multiple paths, modelling trajectories taken by the subject (standing still, walking to the left or right) and the agent (looking straight-ahead or to the left or right). Crucially, only the *gaze* factor is controllable; in other words, the agent can choose where to look based upon her prior beliefs about policies, supplied by their respective expected free energies. The factors generate a discrete outcome in each modality; where each modality is equipped with a likelihood A tensor, whose dimensions correspond to the number of outcomes in each modality times the number of states in each factor. Here, there are eight modalities. The four visual modalities are described in Fig. 3. The remaining four modalities comprise a proprioceptive modality that reports the current direction of gaze, and three auditory modalities with identity mappings from the hidden states that are shared by all agents; namely, *location*, *proximity* and *pose*. The A and B tensors collectively specify the transition priors and likelihoods of the generative model. In hierarchical models there are further tensors linking states at higher levels to the initial states and paths of lower levels. Here, we set these to be uninformative vectors; i.e., no hierarchical constraints. The pseudocode in the panels illustrates the way that these tensors can be specified in terms of straightforward (Boolean) logic.

Here, agents report the location of a subject (e.g., potential predator or mother) in terms of its radial location in an allocentric frame of reference and its proximity (*near* or *far*). In addition, the agents can report whether the subject’s disposition is friendly or not. The requisite inference is difficult (in addition to the limited field of view afforded each agent): first, the agents cannot see motion. This means any movement of the subject has to be inferred from successive inferences about their location. Inferred motion is crucial because it underwrites predictions of location at the next time step, which are broadcast to other agents. Second, the disambiguation between friend and foe is only possible when the subject is *close* to an agent. Fig. 3 describes this setup in terms of what each agent can see, while Fig. 4 describes the hidden states generating observations.

### 3.1 Belief sharing

In simulations of belief-sharing or communication, there is a crucial difference between the way outcomes are generated by the environment and by other agents. Outcomes generated by the environment are caused by hidden states, which are the same for all agents. However, outcomes that underwrite belief-sharing are caused by an agent’s *beliefs* or predictions about hidden states. If belief sharing were implemented by message passing on a factor graph, it would just involve the exchange of (log) posteriors among agents that share beliefs about factor *f*. Expressing this in terms of message passing, just entails supplementing likelihood and prior messages with the corresponding messages from other agents. For agent *n*, this translates into:

(6) $$s_{\tau }^{n,f}=\sigma \left(\mu_{↑A}^{n,f}+\mu_{\to B}^{n,f}+\mu_{\leftarrow B}^{n,f}+\sum_{m\in pa\left(n\right)}(\mu_{↑A}^{m,f}+\mu_{\to B}^{m,f}+\mu_{\leftarrow B}^{m,f})\right)$$

Federated inference of this sort ensures that agents come to share posterior beliefs that minimise the joint free energy over agents (at which point free energy gradients vanish):

(7) $$\begin{array}{c} \frac{\partial F}{\partial s_{\tau }^{n,f}}=\frac{\partial }{\partial s_{\tau }^{n,f}}[s_{\tau }^{n,f}⊙\ell \left(s_{\tau }^{n,f}\right)-s_{\tau }^{n,f}⊙\sum_{m}\left(\mu_{↑A}^{m,f}+\mu_{\to B}^{m,f}+\mu_{\leftarrow B}^{m,f}\right)]=0\\ \Rightarrow \\ \ell \left(s_{\tau }^{n,f}\right)=\sum_{m}\left(\mu_{↑A}^{m,f}+\mu_{\to B}^{m,f}+\mu_{\leftarrow B}^{m,f}\right)\Rightarrow \\ s_{\tau }^{n,f}=\sigma (\mu_{↑A}^{n,f}+\mu_{\to B}^{n,f}+\mu_{\leftarrow B}^{n,f}+\sum_{m\in pa\left(n\right)}\left(\mu_{↑A}^{m,f}+\mu_{\to B}^{m,f}+\mu_{\leftarrow B}^{m,f}\right)) \end{array}$$

This is formally distinct from Bayesian belief updating, in which the likelihood of conditionally independent observations are assimilated. *Belief updating* would result in agents with different posterior beliefs, depending upon their priors. *Belief sharing* assimilates posteriors to evince a consensus that can be likened, anecdotally, to a ‘hive mind’; in which agents inherit both the likelihood *and priors* from other agents. Note that beliefs are only shared when they pertain to the same states of the world, under a shared frame of reference (Fields et al., 2022). This means that only some beliefs are shared (e.g., about the location of a subject in the environment), while others are not (e.g., where each agent is looking).

The direct (peer-to-peer) belief-sharing in (6) may be apt for federated inference, where federated inference can be read as the assimilation of messages from multiple agents during inference or belief updating. However, federated inference—so defined—dissolves the notion of an individual agent. This follows because each agent is defined by its Markov blanket, which precludes reciprocal message passing with other agents (Heins et al., 2023, Palacios et al., 2017, Parr et al., 2020, Pellet and Elisseeff, 2008). In other words, for one agent to be individuated from another agent, they have to be separated by a Markov blanket. This means one agent cannot be a parent (or child) of another; that is, they cannot pass messages to each other. To preserve the conditional independence of agents they have to communicate through a shared Markov blanket; namely, their observations. In sum, to individuate one agent from another requires a (likelihood) mapping from beliefs to exchangeable observations. We will associate the implicit exchange of sufficient statistics with *communication* in general, and language in particular (Isomura et al., 2019).

By equipping each agent with a likelihood mapping from her beliefs to observable outcomes, one effectively creates agents that broadcast their beliefs and recognise what other agents believe. Endowing each agent with an outcome modality $o_{\tau }^{f}$—for each shareable factor—we have, from (5):

(8) $$\begin{array}{c} \mu_{↑A}^{n,f}=\ell (o_{\tau }^{n,f}⊙\mu \left(a^{f}\right))\\ \ell \left(o_{\tau }^{n,f}\right)=\sum_{m\in pa\left(n\right)}\ell (\mu \left(a^{f}\right)⊙s_{\tau }^{m,f})\\ \Rightarrow \\ \mu_{↑A}^{n,f}=\sum_{m\in pa\left(n\right)}\ell \left(s_{\tau }^{m,f}\right):\mu \left(a^{f}\right)=I^{f}\\ =\sum_{m\in pa\left(n\right)}\left(\mu_{↑A}^{m,f}+\mu_{\to B}^{m,f}+\mu_{\leftarrow B}^{m,f}\right) \end{array}$$

Comparison with (6) shows that the ascending likelihood message is formally identical to the log posteriors required for belief-sharing. This holds if the likelihood mappings are identity matrices—and each agent ‘hears’ a probabilistic mixture of outputs generated by other agents. In fact, the likelihood mappings can be any permutation matrix, as we will see later. Notice that the likelihood tensors for broadcasting beliefs are just matrices that uniquely associate an outcome modality with a hidden factor.

In what follows, communication was modelled according to (8), generating a probabilistic outcome (e.g., chorus) heard by all agents.^6^ To keep things simple, we assume that each kind of belief is shared in a separate (e.g., auditory) modality; e.g., ‘word’ or ‘call’. In this setting, each ‘word’ holds more information than conventional tokens because it contains the sufficient statistics over possible outcomes for this kind of word (e.g., encoded in the amplitude of different frequencies of auditory streams). In other words, communication is taken to include not just the content of beliefs but the confidence in those beliefs (Bahrami et al., 2010). A more elaborate simulation of linguistic communication would linearise a word sequence, by equipping the generative model with a lower hierarchical level: e.g., (Friston et al., 2020, Friston et al., 2021). Another approach would be to sample a token in proportion to the (negative) expected free energy of commutative actions (Albarracin et al., 2022, Heins et al., 2023). However, for simplicity, we will deal with the direct exchange of sufficient statistics, necessary for communicating beliefs.

In this setup, an agent’s communication will be imprecise or quiet, if the agent is uncertain about the state in question. Conversely, if the agent has precise beliefs, her contribution will dominate in a way that can be read as speaking loudly and clearly. Note that agents do not hear themselves. Technically, this precludes double-counting of an agent's log posterior: c.f., (Jardri and Deneve, 2013). Neurobiologically, this can be viewed as sensory attenuation; namely, attenuating self-generated sensations (Blakemore et al., 1999, Limanowski, 2017). There are many other models of communication that involve turn-taking and attribution of agency (Friston and Frith, 2015, Ghazanfar and Takahashi, 2014, Wilson and Wilson, 2005): however here, we imagine that the sentinels are constantly reporting their beliefs to each other. This has the particular consequence that the communicated beliefs are posterior predictive distributions over latent states. In other words, the environment generates observations and agents broadcast their beliefs at the same time. This means that the beliefs are based upon observations at the previous time step, and are therefore predictive in nature. In turn, the predictive validity of communicated beliefs rests sensitively on inferred state transitions (e.g., motion of the subject of observation). We will see examples of this below.

### 3.2 Belief sharing and communication

In what follows, we compare belief updating with and without communication, where communication is suppressed by reducing the precision of the auditory likelihood mappings to zero. This means that the agents can neither generate or recognise auditory cues and are effectively rendered incommunicado.

**Fig. 5:**
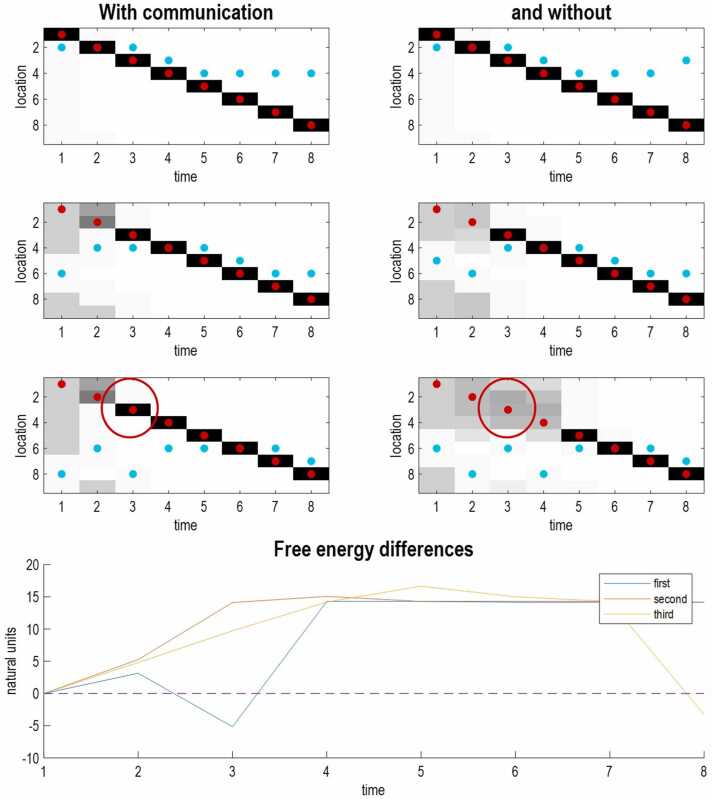
Simulations of active inference. The upper panels depict posterior beliefs—about the location of the subject—as posterior distributions over the nine possible locations (columns) at each of the (eight) timesteps. The right-hand panels reproduce the belief updating shown in the left hand panels but in the absence of communication. Each panel reports the posterior beliefs as a function of time along the x-axis. The beliefs in this case are probability distributions over nine locations, shown as a greyscale image. In other words, uncertain, imprecise beliefs correspond to grey regions that cover multiple locations. Over time, uncertainty is resolved, and beliefs becomes very precise with a concentration of probability in the black regions. The true location is shown by the red dots, while the cyan dots indicate where the agent was looking. Note that these locations are restricted to the agent’s line of sight—and one location to the right and left. The three rows of panels report the belief updating for the three agents. The left panels show belief updating with communication. The three panels on the right show the corresponding active inference in the absence of communication, using the same sequence of hidden states. Communication or language was suppressed by rendering the auditory likelihood mappings very imprecise. The lower panel shows differences in free energy as a function of time for the three agents (coloured lines), when comparing belief updating with and without communication. The red circle highlights the epoch of belief updating illustrated in the next figure. This epoch illustrates the benefits of communication in the sense that the third agent has resolved her uncertainty about the location of the subject by the third epoch, whereas the same agent without communication only forms precise beliefs, after seeing the subject at the fifth epoch.

Fig. 5 illustrates the belief updating and ensuing action for three agents, with and without communication (the left and right panels, respectively). In both conditions, the subject started at the first location and moved clockwise around the agents for eight timesteps (about two seconds of real and simulated time). The most prescient difference, when suppressing communication, is a failure of the second and third agents (second and third rows) to resolve uncertainty about the subject’s location—and act in a suitably pre-emptive fashion. For example, the third agent only forms precise beliefs about location on time step five. In contrast, the third communicating agent quickly infers the location of the subject on the basis of what the first agent says. The first agent sees the subject at the beginning of the episode and broadcasts her precise predictions on the first time step, to which the remaining agents are able to commit; despite not seeing the subject until the third and fifth times steps, respectively.

Note that each agent tries to see the subject by looking towards its inferred location, to the extent that they can, given their limited field of view. This visual tracking is driven purely by expected information gain; namely, the imperative to resolve uncertainty by responding to the epistemic affordances scored by expected free energy: i.e., the information gain in (2). The lower panel of Fig. 5 shows the differences in free energies when comparing the agents with and without communication. With one exception, these differences suggest that communicating agents have a better grip on the world, and a lower free energy. This is most marked for the third agent (yellow line) who only sees the agent in the periphery of her vision on time step five. Interestingly, the first agent appears to have been a little distracted by the second and third agents, with a slightly higher free energy on the third time step.

**Fig. 6:**
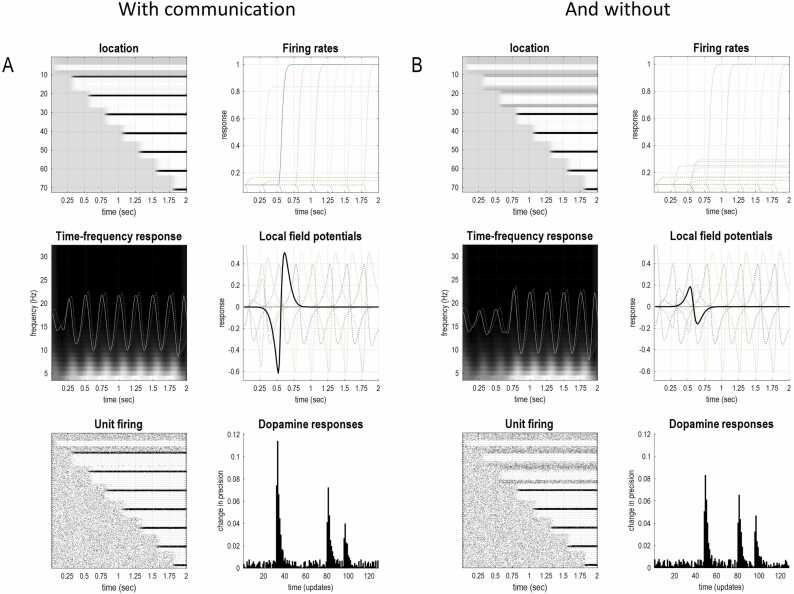
Simulated electrophysiological responses. This figure shows expectations about hidden states for the third agent with (A) and without (B) communication. A: The upper left panel shows the activity (firing rate) of units encoding the location of a subject over time points (i.e., epochs), each corresponding to roughly 250 ms of simulated and computational time. These responses are organised such that the upper rows encode the probability of alternative states in the first epoch, with subsequent epochs in lower rows. In other words, the top row shows the expectations about hidden states at the beginning of the exchange—and how these expectations evolve over time. Conversely, the first column shows expectations about the future. The plot to the right of the image presents the same information to illustrate the implicit evidence accumulation. These values can be interpreted as neuronal firing rates of units encoding expectations. The associated local field potentials (i.e. the rate of change of neuronal firing) are shown in the middle panels in terms of a time frequency decomposition (left middle) and local field potentials (right middle). The local field potential showing the greatest difference between the two conditions is highlighted in black. The lower left panel reproduces the upper left panel but in the form of a raster plot, assuming the expectations have a Poisson rate code. Similarly, the lower right panel shows the simulated dopaminergic responses under a Poisson rate code assumption. Dopamine, in this formulation, scores changes in the confidence afforded policy selection; namely, the negative entropy of posterior beliefs over combinations of actions based upon expected free energy. In this example, the agent is more confident about her action (i.e., where to look) when she is able to communicate with other agents. This is manifest as a short latency, high amplitude, phasic dopaminergic response.

Fig. 6 shows the simulated neuronal responses that would accompany the belief updating illustrated in Fig. 5. These synthetic results are shown to underscore the fact that the variational message passing described above can be implemented in a neuronally plausible fashion (Friston et al., 2017a, Friston et al., 2017b, Parr and Friston, 2018), producing simulated electrophysiological responses that are similar to those observed empirically. In this example, the key takeaway is the greater degree of belief updating when an agent can assimilate informative communications (left panels), relative to when she has an imprecise auditory likelihood mapping (right panels). An informative likelihood mapping enables auditory input to resolve uncertainty about latent states, producing a greater degree of belief updating and simulated electrophysiological responses.

The accompanying resolution of uncertainty is scored by simulated dopaminergic discharges in the lower right panels that accompany an augmented event-related potential (middle panels). Please see (Friston et al., 2017a, Friston et al., 2014, Friston et al., 2017b, Parr and Friston, 2018) for a fuller discussion. This biomimetic formulation of variational message passing replaces the fixed point iteration scheme in Fig. 3 with a gradient descent on variational free energy. Neuronal implementation is not a central part of the current argument; however, it speaks to the sort of predictions that can be made, when testing process theories based upon active inference.

In summary, this section has showcased the role of belief-sharing through communication in resolving uncertainty during active inference. In effect, it enables communicating agents to benefit from the complementary perspectives and observations afforded each agent (Bahrami et al., 2010). In the next section, we turn to active learning and the acquisition of the likelihood mapping that underwrites the generation and recognition of communicative exchanges.

## 4 Active inference and learning

" The second question is why, out of the infinite range of knowable items in the universe, certain pieces of knowledge are more ardently sought and more readily retained than others" (Berlyne, 1954) p180.

In this section, we turn to active learning and the acquisition of an apt likelihood model for communication. Active learning has a special meaning in this context. It implies that the action that updates the Dirichlet counts (c.f., experience-dependent plasticity) is selected on the basis of expected free energy; where expected free energy—from the perspective of model parameters—is, effectively, the mutual information encoded by the Dirichlet tensors: see Eq. (3). Put simply, this means that an update—to knowledge encoded in likelihood tensors—is only selected in proportion to the expected information gain. Consider two policies: to update or not to update. From Fig. 2, we have (dropping the modality superscript for clarity):

(9) $$\begin{array}{c} \Delta a=o_{\tau }{⊗}_{i\in pa}s_{\tau }^{i}\\ E_{Q}\left[a\right|u=u_{o}]=a|u_{o}=a\\ E_{Q}\left[a\right|u=u_{1}]=a|u_{1}=a+\Delta a\\ \bar{a}=:\frac{a}{\Sigma \left(a\right)}=E[P(o,s\left|a\right)] \end{array}$$

Here,$\Sigma (a)=:1⊙a{⊙}_{i\in pa}1$ is just sum of all tensor elements. The prior probability of committing to an update is given by the expected free energy of the respective Dirichlet parameters, which scores the expected information or gain (i.e., mutual information) and cost^7^:

(10) $$\begin{array}{c} P\left(u\right)=\sigma (-\alpha \cdot G(a\left|u\right))\\ G\left(a\right)=-\underset{\mathrm{mutual}\;\;\mathrm{information}}{\underset{⏟}{E_{Q_{a}}[D_{KL}[Q(s,o\left|a\right)||Q(s\left|a\right)P(o\left|a\right)]]}}-\underset{\mathrm{expected}\;\;\mathrm{cost}}{\underset{⏟}{E_{Q_{a}}\left[\;\ln\;P(o\left|c\right)\right]}}\\ =\left(1⊙\overset{¯}{a}\right)⊙\ell \left(1⊙\overset{¯}{a}\right)+\left(\overset{¯}{a}⊙1\right)⊙\ell \left(\overset{¯}{a}⊙1\right)-1⊙\left(\overset{¯}{a}\times \ell \left(\overset{¯}{a}\right)\right)⊙1-\varphi \left(c\right)⊙\left(\overset{¯}{a}⊙1\right) \end{array}$$

This prior over update policies furnishes a Bayesian model average of the likelihood parameters, effectively marginalising over update policies:

(11) $$\begin{array}{c} E_{Q}\left[a_{\tau +1}^{g}\right]=P\left(u_{0}\right)\cdot E_{Q}\left[a_{\tau }^{g}\right|u=u_{o}]+P\left(u_{1}\right)E_{Q}\left[a_{\tau }^{g}\right|u=u_{i}]\\ \Rightarrow \\ a_{\tau +1}^{g}=P\left(u_{0}\right)\cdot a_{\tau }^{g}+P\left(u_{1}\right)(a_{\tau }^{g}+\Delta a_{\tau }^{g})\\ =a_{\tau }^{g}+P\left(u_{1}\right)\Delta a_{\tau }^{g} \end{array}$$

In Eq. (10), α plays the role of a hyperprior that determines the sensitivity to expected free energy. When this precision parameter is large, the Bayesian model average above becomes Bayesian model selection; i.e., either the update is selected, or it is not. It may seem odd to impose constraints on updates in this fashion; however, active inference rests on a circular causality, in which actions on the world realise predicted outcomes. This means that committing to a world model, with precise likelihood mappings, can bring about the precise generation of outcomes. Communication—and implicit niche co-construction—is a nice example of this, as we will see later.

The active learning in (11) would be Bayes optimal in a world that never changed; enabling the eternal accumulation of Dirichlet counts, such that more and more evidence would be required to change the expected likelihoods. However, when the world is changing (e.g., through the action of agents that themselves are learning) there exists a particular timescale over which evidence should be retained. One can accommodate this by introducing a hyperprior on the effective number of observations that should be retained as follows; noting that the total Dirichlet counts—that could be accumulated after each observation— sum to one, by construction:

(12) $$\begin{array}{c} a_{\tau +1}^{g}=(a_{\tau }^{g}+P\left(u_{1}\right)\Delta a_{\tau }^{g})\left(\frac{\eta }{\eta +P\left(u_{1}\right)}\right),\;\;\Sigma (\Delta a_{\tau }^{g})=1\\ \Rightarrow \\ \eta (\Sigma \left(a_{\tau +1}^{g}\right)-\Sigma \left(a_{\tau }^{g}\right))=P\left(u_{1}\right)(\eta -\Sigma \left(a_{\tau +1}^{g}\right))\\ \Sigma \left(a_{\tau +1}^{g}\right)=\eta \Rightarrow \Sigma \left(a_{\tau +1}^{g}\right)=\Sigma \left(a_{\tau }^{g}\right) \end{array}$$

Eq. (12) says the total number of Dirichlet counts saturates at η. In other words, the Dirichlet counts acquire an upper bound, via a slight decay at the point of updating. Technically, this can be regarded as a hyperprior that implements Bayes-optimal forgetting in a volatile environment (Ishii et al., 2002, Moens and Zenon, 2019). On this view, η is the timescale over which an adiabatic approximation holds. Neurobiologically, this is not unrelated to reconsolidation of memories (Stickgold and Walker, 2007) and synaptic homeostasis (Huber et al., 2004, Toutounji and Pipa, 2014).

Heuristically, the hyperprior determines how impressionable the agent is; in the sense that if the number of Dirichlet counts is small, new observations will have a greater effect on the expected likelihood (because their relative values change more readily). In our simulations, all the agents were effectively young and impressionable with η set to 32. In other words, the last 32 experiences predominate in the accumulation of knowledge or memory encoded in the Dirichlet parameters. Notice how learning has become a key part of inference, with its own dynamics. This reflects the fact that—in active inference—there are probability distributions over all random variables; including hidden states, model parameters and their structure. This enables the application of variational Bayes to simulate or realise (Bayes-optimal) behaviour.

### 4.1 Synthetic learning

To illustrate active learning in the current setting, we simulated the acquisition of language by a child of one agent, who sees and hears the same things as her parent but has a completely ambiguous or imprecise likelihood mapping. This means the child can neither understand, nor participate, in communication, until she has learned a sufficiently precise auditory mapping. This naïve agent can be thought of as a child in virtue of the fact that her prior Dirichlet counts were small (uniformly one), meaning that she is inherently impressionable.

**Fig. 7:**
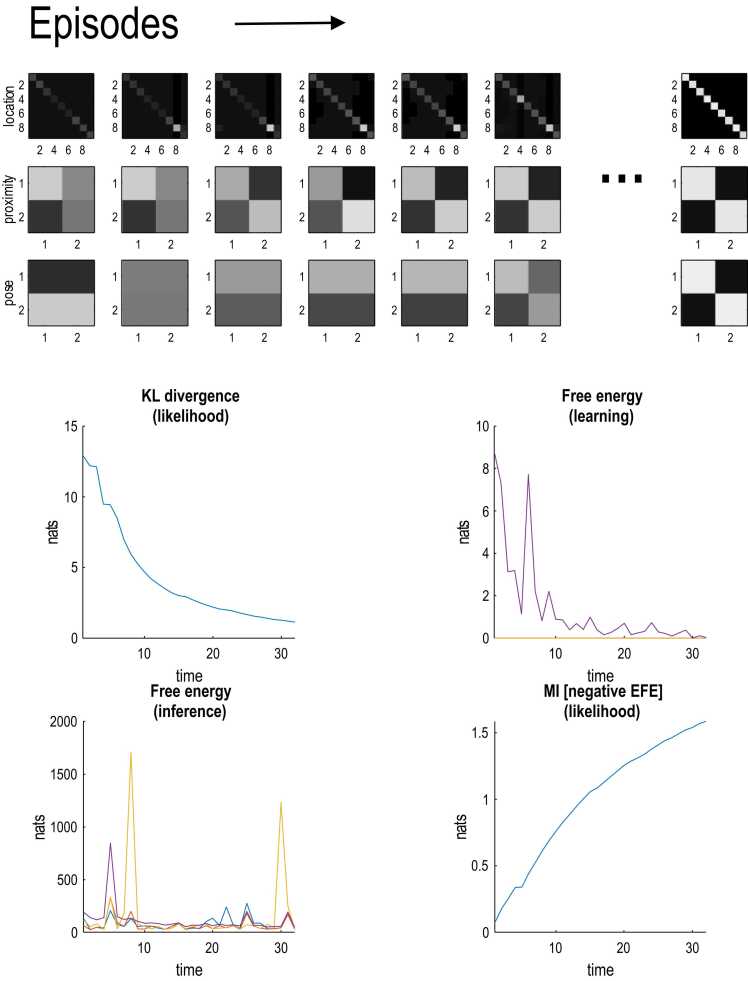
language acquisition. this figure illustrates the acquisition of language in terms of the (three) auditory likelihood mappings, starting from a completely naïve or uniform tensor (with all prior Dirichlet counts set to 1) to a structured identity matrix, after a sufficient number of exposures; here, 32 episodes. Each episode lasted for 16 timesteps, where each time step corresponds to 1/4 of the second (250 ms). The upper panels show the likelihood mappings for the three auditory modalities (reporting *location*, *proximity* and *pose*) over the first six and last exposures. The lower panels report the subsequent learning in terms of various free energies and relative entropies (a.k.a., KL divergences). The upper right panel shows the KL divergence of the likelihood mapping (summed over hidden states and outcome modalities) between the child and her parent. It can be seen that this falls systematically during language acquisition. The lower left panel shows the accompanying free energy of inference (i.e., due to posterior beliefs) for the three agents over time. The instances of high free energy correspond to episodes in which there is very little (and possibly no information) about one or more latent states for one or more agents. The upper right panel scores the free energy associated with learning. This is the information gain as measured by the KL divergence between the likelihood mappings before and after parametric belief updates. It can be seen that this is greatest during early periods of exposure and then converges to zero as precise likelihood mappings are acquired. The lower right panel shows the increase in the mutual information of the likelihood mappings as a function of acquisition time. This is equivalent to the negative expected free energy due to the model (i.e., Dirichlet) parameters, noting that in these simulations the expected cost was zero.

Fig. 7 shows the acquisition of language by the child of the first agent. The upper panels show the emergence of a precise likelihood mapping, as Dirichlet parameters are accumulated by seeing and hearing the same thing as her parent. In this example, there were 32 exposures or episodes—of 16 epochs—by which time the learning of a precise language mapping was almost complete—as reflected in the small KL divergence between the child and her parent, and the high mutual information encoded by the respective auditory likelihood mappings (lower panels in Fig. 7). Note that some episodes were informative, and others were not—as reflected in the episodic variations in free energy associated with inference (lower left panel). This reflects the fact that in some trials there was no information about pose (i.e., *friend* or *foe*), unless the subject approached one of the agents. These results illustrate language acquisition and pave the way for a simple simulation of how language can be transmitted over generations.

**Fig. 8:**
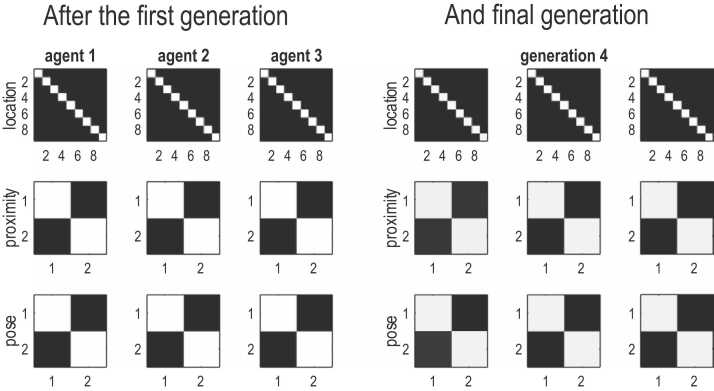
transgenerational transmission. This figure shows the likelihood mappings pertaining to language or auditory modalities for three parents at the beginning of the first generation (left panel) and after all the parents have been replaced by their children in the fourth generation (right panel). Each column corresponds to an agent and each row corresponds to a likelihood mapping (shown in image format) from hidden states 23 outcome modalities (location, proximity and pose). These likelihood mappings correspond to the final mappings—after learning—shown in Fig. 7 (on the far right). The key thing to notice here is that these likelihood mappings are virtually indistinguishable; despite the fact that subsequent generations had to learn these mappings from scratch.

After 32 episodes, the child replaced her parent and the next parent in line was given a new child. This process was repeated until all parents had been replaced by children. The auditory likelihood mappings encoding the generation and recognition of language are shown in Fig. 8. With the exception of slightly imprecise auditory mappings for proximity and pose, these mappings are almost identical to those of the parents. In other words, after four generations, we end up where we started. The remarkable thing here is that language acquisition was mediated entirely through experience-dependent plasticity and active learning. At no point were the prior Dirichlet parameters or structure of any child informed about the language used by her parents. From an evolutionary perspective, cultural transmission—mediated by experience-dependent plasticity of a (neuro) developmental sort—could be read in terms of niche construction and evolutionary developmental biology (Ghazanfar and Takahashi, 2014, Hauser et al., 2002, Heyes, 2018, Laland et al., 1999, Lehmann, 2008, Vasil et al., 2020, Veissiere et al., 2019). Given that the simulations only covered about two minutes of simulated (and real) time, one could regard them as simulating the learning of a new videogame.

In the previous section, we saw that precise likelihood mappings were necessary for communication. In this section, we saw that agents acquire precise likelihood mappings, because this is what they expect to acquire a priori. This begs the question: does communication emerge from prior expectations? The next section addresses this question by starting with three agents devoid of any language capabilities.

## 5 Active inference and selection

In this section, we turn to the emergence of language as a consequence of nested, free-energy minimising processes. In these numerical experiments, we rendered all the auditory likelihood mappings imprecise (and learnable) by setting all the Dirichlet parameters to one, plus an unsigned random Gaussian variate. We then exposed the three agents to 512 episodes to see if language mappings—and attending communication—would emerge.

**Fig. 9:**
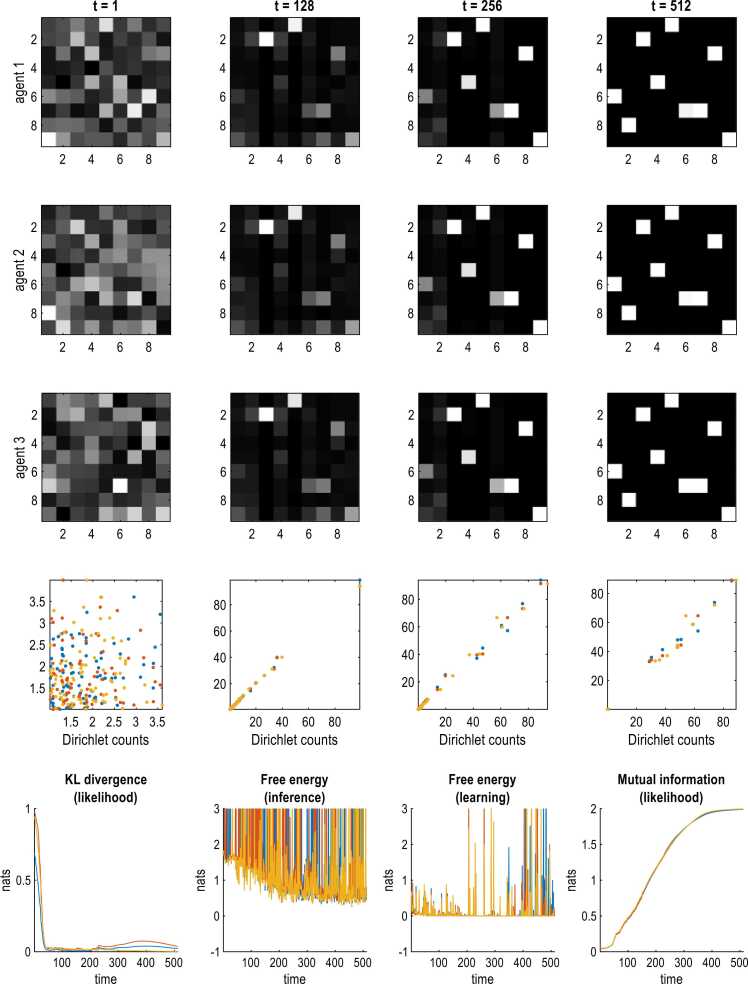
the emergence of language. This figure uses a similar format to Fig. 7; however, in this simulation we exposed three language-naïve agents to 512 episodes. This means the likelihood mappings start off with a random configuration and autodidactically converge to each other. This contrasts with Fig. 7 in which a naïve agent converged to an (identity) likelihood mapping by learning from her teachers. The upper panels show the likelihood mapping from hidden states pertaining to location and the corresponding auditory outcome, for three agents (in each column). The left columns report the initial random connectivity that subsequently resolves into a sparse structure, with repeated exposure, indicated by the number of exposures, *t*. Crucially, a few precise mappings emerge by 128 episodes that are subsequently refined. By the end of the simulation, nearly every hidden state has become associated with a unique ‘word’ in a way that is shared over agents. Interestingly, locations 5 and 6 are reported with the same ‘word’ (outcome 7), speaking to a slight coarsening of the language used by the agents in Fig. 7. The plots in the penultimate row show the increasing correlation between the corresponding Dirichlet parameters for all pairs of agents. Initially, there are no correlations because the initial parameters (c.f., connections) were assigned randomly by adding an unsigned random Gaussian variates to 1, for every connection. After 128 exposures, a few of these connections have been selected and acquire high Dirichlet counts of about 80. As time proceeds, other connections are selected, while the remaining connections are eliminated; rendering the final connectivity sparse. The lower row illustrates the correlates of this structure learning or language acquisition in terms of the KL divergence between all pairs of likelihood mappings between the agents; the ensuing variational free energy associated with inference; the free energy associated with learning and, finally, the mutual information of the likelihood mappings for location, for each agent (coloured lines). In this example, the likelihood mappings become nearly identical by about 128 exposures with a reduction in variational free energy. As anticipated, there is a progressive increase in the mutual information afforded by these auditory likelihood mappings, saturating at about two natural units (i.e., about three bits).

The results are shown in Fig. 9 and speak to a fairly rapid and precise emergence of a shared language that, interestingly, was distinct from the language of the agents in the preceding simulations. In other words, the meaning of various ‘words’ was completely different, such that one of the agents in Fig. 9 would not be able to communicate with the agents in Fig. 8. This convergence to a common ground (Allan, 2013, Tomasello, 2016) or frame of reference (Fields et al., 2021a, Fields et al., 2021b) was mediated by free energy selection processes at the level of the model structure, using Bayesian model reduction (Smith et al., 2020).

### 5.1 Bayesian model reduction and structure learning

In contrast to learning—that optimises posteriors over parameters—Bayesian model selection or structure learning (Tenenbaum et al., 2011, Tervo et al., 2016, Tomasello, 2016) can be framed as optimising the *priors* over model parameters. Bayesian model reduction is a top-down approach to this kind of structure learning, which starts with an expressive model and removes redundant parameters to reveal the best sparsity structure. Crucially, Bayesian model reduction can be applied to the posterior beliefs after the data have been assimilated. In other words, Bayesian model reduction is a post hoc optimisation that refines current beliefs based upon alternative models that may provide potentially simpler explanations (Friston and Penny, 2011).

Technically, Bayesian model reduction is a generalisation of ubiquitous procedures in statistics, ranging from the Savage-Dickey ratio (Savage, 1954), through to classical F-tests. In our context, it reduces to something remarkably simple: by applying Bayes rules to full and reduced models it is straightforward to show that the change in free energy can be expressed in terms of posterior Dirichlet counts **a**, prior counts *a* and the prior counts that define a reduced model *a*’. Using Β to denote the beta function, we have (Friston et al., 2018):

(13) $$\begin{array}{c} \Delta F=\;\ln\;P(o\left|a\right)-\;\ln\;P(o\left|a\prime \right)\\ =\;\ln\;Β\left(a\right)+\;\ln\;Β\left(a\prime \right)-\;\ln\;Β\left(a\right)-\;\ln\;Β(a+a\prime -a)\\ a\prime =a+a\prime -a \end{array}$$

Here, a′ corresponds to the posterior one would have obtained under the reduced priors.

Clearly, to realise this form of free energy minimisation, one has to have a space of models or reduced priors to evaluate. So, how does one explore the space of priors over parameters? The idea behind active model selection is to consider priors that minimise expected free energy. These models entail a high mutual information and sparse probabilistic mappings: c.f., (Navarro and Perfors, 2011). The active model selection considered here entertains some new log priors^8^ that decrease expected free energy by one natural unit (omitting superscripts for clarity):

(14) $$\begin{array}{c} \ell \left(\overset{ˆ}{a}\right)=\ell \left(a\right)-\frac{\partial G\left(a\right)}{\partial \ell \left(a\right)}\Rightarrow \overset{ˆ}{a}=a\times \exp\left(-a\times \frac{\partial G}{\partial a}\right)\\ \frac{\partial G}{\partial a}=\frac{\partial \overset{¯}{a}}{\partial a}\times \frac{\partial G}{\partial \overset{¯}{a}}=\left(\frac{1}{\Sigma \left(a\right)}-\frac{\overset{¯}{a}}{\Sigma \left(a\right)}\right)\times \frac{\partial G}{\partial \overset{¯}{a}}\\ \frac{\partial G}{\partial \overset{¯}{a}}=1-\ell \left(\overset{¯}{a}\right)+\ell \left((\overset{¯}{a}⊙1)⊗(1⊙\overset{¯}{a})\right)-\varphi \left(c\right)⊗1 \end{array}$$

As in active learning, one can then use Bayesian model averaging to take a weighted mixture of old and new (reduced) priors:

(15) $$\begin{array}{c} E_{P}\left[a_{\tau }^{g}\right|u=u_{o}]=a\\ E_{P}\left[a_{\tau }^{g}\right|u=u_{1}]=\overset{ˆ}{a}\\ E_{P}\left[a_{\tau }^{g}\right]=P\left(u_{0}\right)\cdot E_{P}\left[a\right|u=u_{o}]+P\left(u_{1}\right)\cdot E_{P}\left[a\right|u=u_{1}]\\ \Rightarrow \\ E_{P}\left[a_{\tau }^{g}\right]=a\prime =P\left(u_{0}\right)\cdot a+P\left(u_{1}\right)\cdot \overset{ˆ}{a}\\ E_{Q}\left[a_{\tau }^{g}\right]=a\prime =a+a\prime -a\\ P\left(u\right)=\sigma (-\alpha \cdot F\left(u\right)),F\left(u\right)=[0,\Delta F] \end{array}$$

Here, the implicit Bayesian model averaging weights each model by its evidence as scored by the reduction in variational free energy. This kind of model selection is therefore guaranteed to select structures with precise or unambiguous probabilistic mappings. When deployed in the simulations of language emergence, the auditory likelihood mappings converge to the same precise structure over agents; thereby enabling the communication illustrated in Fig. 9.

Note how active learning and selection complement each other. In active learning, posterior parameters change to minimise variational free energy if, and only if, expected free energy is reduced. Conversely, in active selection, prior parameters change to minimise expected free energy if, and only if, variational free energy is reduced—as scored with Bayesian model reduction. The implicit bootstrapping underwrites self-evidencing towards precise and predictable exchanges with—in the context of communication—a co-constructed world.

**Fig. 10:**
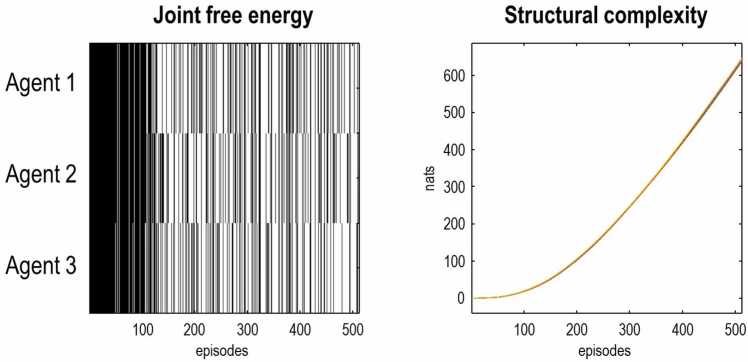
Schematic. the emergence of structural complexity. The left panel indicates episodes in which the variational free energy of inference—per modality and observation—fell below one (white bars), towards its lower bound of zero, for each agent (rows). This illustrates the incidence of low free energy (high model evidence) episodes increases as language is acquired. The right panel shows the increase in structural complexity that underwrites this improvement in inference—mediated by belief sharing or communication—over time. The three lines correspond to the three agents. This is the cumulative expected free energy due to learning in Fig. 9.

Fig. 10 shows the ensuing increase in the ability of agents to resolve uncertainty about the scene. The left panel illustrates episodes when variational free energy, per epoch and modality, was less than one. In other words, when the self-information or surprisal afforded by each observation was negligible (Kass and Raftery, 1995).^9^ Initially, all three agents remain rather confused about what is going on around them, with high levels of variational free energy. As they acquire the ability to share their beliefs, the number of episodes with a low free energy starts to increase where, in some instances, all three agents have a veridical understanding of the latent states generating their observations. This is accompanied by a progressive sparsification (Spielman and Srivastava, 2011) and convergence of their likelihood mappings onto a shared structure. The complexity of this structure can be scored in terms of the cumulative KL divergence between the posterior over the Dirichlet distributions, in relation to the initial distributions (i.e., an approximation to the information length or distance between initial and current posteriors over parameters). This is the cumulative free energy or information gain (i.e., complexity) due to parameters and can be read a measure of *structural complexity* that increases progressively with experience (right panel of Fig. 10**).**

### 5.2 Supervised structure learning

When illustrating active inference, we saw that belief-sharing augmented the resolution of uncertainty, enabling a more precise grip on states of affairs (see Fig. 5). One can now ask whether belief sharing has a similar synergetic role in learning. In other words, could communication scaffold the learning of likelihood models and thereby disentangle observations to recognise their hidden causes? To address this, we repeated the preceding simulations, equipping all agents with precise language mappings but withholding precise visual mappings from the last agent (by setting all the Dirichlet parameters to one, plus an unsigned random Gaussian variate). Heuristically, this can be regarded as replacing an experienced sentinel with a young novice, who has yet to develop a likelihood model for the causes of her visual observations. However, she can hear her ‘supervisors’ talking about those causes. Heuristically, this can be likened to teaching a child to read by pairing the presentation of pictures and spoken names of an object.

**Fig. 11:**
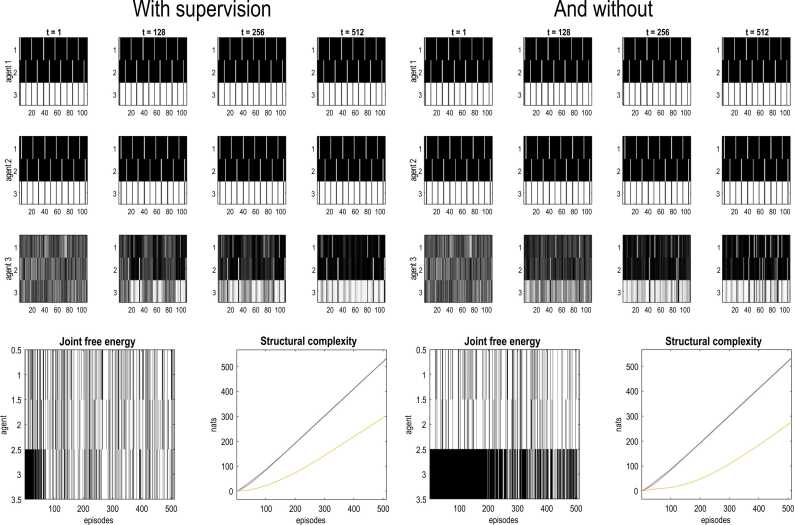
**supervised structure learning**. This graphic reports numerical experiments asking whether communication enhances the learning of likelihood models. In this example, the last agent was rendered visually naïve by setting the Dirichlet counts mapping from hidden states to visual outcomes to one (plus an unsigned random Gaussian variate). However, proprioceptive and auditory (identity) mappings were preserved for all three agents. The ensuing simulations illustrate learning of the visual likelihood mapping: e.g., the development of visual pathways and subsequent skilled recognition of the causes of visual sensations. The left hand panels illustrate the implicit supervised learning, while the right hand panels reproduce the same stimulation in the absence of supervision (by rendering the third agent impervious to auditory input). The upper panels show the likelihood tensor mapping from hidden states to the central contrast energy modality for the three agents (rows), over selected time points during the 512 simulated episodes (columns). Because these likelihood mappings are tensors, we have placed the slices of the tensor next to each other, to form a matrix. For example, the first row of each matrix illustrates the fact that there are six combinations of hidden states generating a *near* outcome. These combinations correspond to the three directions of *gaze* times the two *poses* of the subject (friend or foe). The lower panels report the ensuing inference and cumulative free energies due to learning (i.e., structural complexity) using the format of Fig. 10. The key point to take from this figure is that supervision—via belief sharing—enables a grip on the world that is indistinguishable from agents with veridical generative models. Conversely, when denied supervision, learning is based purely upon visual and proprioceptive inputs. In this instance, the unsupervised agent learns slowly, failing to disentangle the causes of her observations and attain the free energy minimisation of her conspecifics. This is reflected in a slight lower structural complexity of her likelihood mappings, which are more disorganised and diffuse with, implicitly, a lower mutual information.

Under this kind of supervised structure learning, the supervisee quickly comes to learn a visual likelihood mapping—that is sufficient to render her inferences indistinguishable from her supervisors—by about 64 exposures. Fig. 11 illustrates this in terms of the acquisition of the likelihood tensors mapping from hidden states to visual outcomes (here, the central, contrast energy modality). This supervised structure learning can be contrasted with the learning in the right panels of Fig. 11, when the novice cannot hear her supervisors (implemented by setting the Dirichlet counts of her auditory likelihood mappings to 64 everywhere). In the absence of supervision, learning proceeds slowly and does not support skilled inference, even after 512 exposures.

These numerical analyses speak to a generic point: belief-sharing appears to be a fundamental in realising joint free energy minimisation, distributed over ensembles of free energy minimising processes and over the timescales at which these processes unfold. Technically, this is just a realisation of a variational principle of least action, where action is the path or time integral of distributed—and extensive^10^—variational free energies.

There are clearly many other simulations that we could pursue from this point. For example, we could combine the above simulations to simulate evolution by selecting those agents that have the lowest free energy (i.e., highest marginal likelihood or adaptive fitness) to augment the structure learning and language acquisition evinced above (Friston et al., 2023a). One can also consider the emergence of language under different frames of reference. For example, we have assumed that the generative model of each agent shares a common frame of reference in relation to allocentric locations. This is not necessary. In principle, it should be possible to use hidden states in an egocentric frame of reference and have agent-specific likelihood mappings that support belief sharing. This kind of simulation would become even more interesting if the agents moved around. However, for the purposes of the current paper, we now turn to a discussion of the phenomena illustrated by these numerical studies.

## 6 Discussion

The foregoing illustrates the emergence of distributed (i.e., federated) inference and learning—where posterior beliefs are shared among agents—under the imperative to maximise the evidence for (generative) models of a shared world; namely, self-evidencing (Hohwy, 2016). This can be read from a number of perspectives: from a systemic perspective, this emergence can be read as the minimisation of joint free energy that ensues when inference, learning and selection are simulated as nested free energy minimising processes. An anthropomorphic interpretation is in terms of linguistic communication; namely, the acquisition of language, and its transmission over generations, in the spirit of cultural niche construction.

A key technical point illustrated in these simulations is the optimisation of free energy functionals of (Bayesian) *beliefs* about the latent causes of observations over temporal scales. This foregrounds the importance of working with probabilistic representations of latent states, parameters and structures that constitute a generative world model. Active learning and model selection would not be possible without the sufficient statistics of beliefs about model parameters and implicit model structure. When working with discrete state space models, the requisite variational updates transpire to be straightforward, local and—at a certain level of analysis—biomimetic. In what follows, we consider some key perspectives on the ensuing mechanics.

### 6.1 Self organisation: a perspective from physics

It is interesting to reflect upon the behaviours that emerge—under free energy minimisation—in light of questions about emergence in random dynamical systems (Arnold, 2003, Crauel and Flandoli, 1994); namely, is the emergence of sparse coupling and generalised synchrony inevitable? (Bak et al., 1988, Ellis et al., 2011, Ellison et al., 2011, England, 2013, England, 2015, Gershenson, 2012, Hunt et al., 1997, Jafri et al., 2016, Jeffery et al., 2019, Namikawa, 2005, Sakthivadivel, 2022b). In other words, is the sparse coupling, self-organised criticality and synchronisation of chaos—seen in complex dynamical systems—a necessary property of such systems that exist?

As noted in the introduction, the free energy principle just prescribes a variational principle of least action that can be applied to any random dynamical system that possesses an attracting set (i.e., a pullback attractor) with a Markov blanket (Crauel and Flandoli, 1994, Friston et al., 2022a, Sakthivadivel, 2022b). This means that the accompanying dynamics of such systems conform to certain principles that can either be articulated in terms of the free energy principle or, equivalently, a constrained maximum entropy principle (Sakthivadivel, 2022a). The constraints in question here are furnished by the generative model that describes the pullback attractor in in terms of a probability density. In effect, this enables one to simulate a system—as it converges on its pullback attractor—by expressing the dynamics as a functional of a generative model. The numerical analyses above are an example of this, under generative models of discrete states.

This formulation licences teleological interpretations, such as optimisation, inference and learning. Indeed, when variational and expected free energy are read as objective functions, they can be seen as equivalent to Bayes-optimal inference and learning, respectively (Winn and Bishop, 2005). However, one ubiquitous aspect of free energy minimisation does not have a clear teleology. This is the emergence of complexity as free energy is minimised at various timescales. Heuristically, this is unremarkable in the sense that free energy is a bound on log evidence. And log evidence is accuracy minus complexity.^11^ This means that if any system or agent learns a more accurate account of its exchanges with an environment (or other agents) its complexity must increase.

What is remarkable is the way in which this increasing complexity is expressed across temporal scales in the numerical studies above. While, on average, the variational free energy associated with beliefs about latent states declines, with learning and language acquisition, the complexity associated with the likelihood parameters increases, as knowledge is accumulated.^12^ This means that the agents or observers progressively evince a more complex and sparse internal structure—quantified, for example, in the structural complexity of Fig. 10. The question now is whether this kind of structural complexity goes hand in hand with communication. In other words, are communication and structural complexity themselves emergent properties of any loosely-coupled random dynamical system within which subsystems (e.g., agents) can be identified. Put simply, are our generative or world models destined for progressive increases in complexity simply because we have to explain and understand our exchange with conspecifics, colleagues, confederates and conspirators. Clearly, to answer this kind of question with simulations one will have to scale up the numerical studies above. Some (active inference) work in this direction addresses the spread of ideas at scale (Albarracin et al., 2022, Heins et al., 2023, Kastel and Hesp, 2021).

### 6.2 Conversely, communication begets space: a perspective from quantum information theory

The communication considered above unfolds in space and time; indeed, location in space is what it is primarily about. The communicating agents are both embedded in space and separated in space. The way that the agents are embedded in space not only gives them something to talk about; it also assures that they have different points of view, and hence that they only partially share their world: c.f., (Williford et al., 2018). All of this is so obvious that it is seldom thought about. Distinct agents occupying distinct positions in a surrounding spatial ‘container’ is assumed as a matter of course when modeling communication.

The model presented in Fig. 1, Fig. 2, however, makes no assumptions about an embedding space: it is constructed entirely out of states (which occupy some state-space) and tensors that act on them (operators on the state-space): c.f., (Fields et al., 2023a, Knill and Laflamme, 1997). The idea of an embedding space that supports a radial distance and a distinction between angles of view is not introduced until Fig. 3. We can therefore ask: where does this (projective) embedding space come from? Is it just an assumption of convenience? What role is the embedding space playing in the communication scenario, other than providing something (location) for the observers to talk about?

Fig. 3 makes the second role of the embedding space explicit: it serves to distinguish and separate the agents. The embedding space is not, however, strictly needed for this; the model could simply have assumed that the agents all have mutually conditionally independent states (i.e., that each agent has her own Markov blanket). In this case, Fig. 3 can be described as depicting a set of distinct agents that share a classical communication channel. The assumption of a shared generative model then becomes the assumption that they share a ‘language’ that allows them to make sense of—i.e., assign sufficiently similar semantics to—each other’s messages. The agent’s perceptual capabilities are described in spatial terms, in terms of “close” versus “near” detectors and angle of gaze. These can, however, also be considered abstract variables—a one-bit variable and a two-bit variable—to which their shared semantics refers. The computation each agent performs remains the same: correlating the values of these variables that they obtain by direct interaction with the world with the values that they obtain from the channel, and hence from the (otherwise unperceived) other agents.

The results of the numerical experiments can now be re-interpreted. What the agents discover is that they share an error-correcting code: there is something redundant about their shared world that confers particular symmetries on the correlations between what they ‘see’ and what they ‘hear’. With sufficient experience, each can infer that there is a fast, almost always cyclic permutation symmetry that is coupled to a slower binary oscillation. They share, in other words, a two-dimensional world with an angular and a radial degree of freedom. As noted by one of our reviewers, these arguments might generalize to any metric space that underwrites sense making and communication: “To me, the question here is more general, about the need for a commonly experienced ground, world, spaces in general, allowing a common system of beliefs, and language [to share messages] about it.”

The idea that communication and space are effectively dual concepts—tied together by the ability of each to function as an error-correcting code for the other—is fundamental to much of quantum gravity research (Bain, 2020, Fields et al., 2023b). It is also attested by the co-development of sensory integration and the sense of space via motor babbling in infants (Baranes and Oudeyer, 2009, Saegusa et al., 2009). That relatively simple experiments, motivated by the free energy principle, illuminate this deep connection is surely of interest.

### 6.3 Federated learning and distributed cognition: a perspective from computer science

In computer science, distributed machine learning has become increasingly important (Verbraeken et al., 2020), especially in the context of deep learning: recent breakthroughs in training large language models (Biderman et al., 2023) or agents with deep reinforcement learning (OpenAI et al., 2019) are fueled by running the training process on hundreds of GPUs in parallel. In these systems, the optimization method used is stochastic gradient descent (SGD), where parameter updates are calculated by averaging the gradients over a batch of data. Parallelism can hence be exploited by duplicating the model over a number of workers—each calculating a gradient update on a part of a [mini]batch, which is then broadcasted to the others: i.e., data parallelism (Goyal et al., 2017). A different way for parallelizing the training workload is by exploiting the layered structure of deep neural networks, and deploying different layers on different workers: i.e., model parallelism (Shoeybi et al., 2019).

Federated machine learning is a special case of distributed machine learning (Yang et al., 2019), in which the training process is distributed among multiple parties, each with access to its own dataset. Crucially, each party wants to learn the better model without, however, sharing their raw data, e.g., due to privacy or security concerns. When applied to distributed SGD, each party calculates gradients locally on parts of their dataset and shares only these with other parties, e.g., further secured by using secret sharing (Bonawitz et al., 2017), homomorphic encryption (Phong et al., 2018), or differential privacy (Shokri and Shmatikov, 2015).

The processes illustrated in this paper speak to a more liberal notion of federated learning. First, instead of limiting belief-sharing to a noisy gradient estimate—from a random minibatch of data in deep learning—under active inference, agents communicate full belief distributions. This renders communication more effective, as information is only shared when required, i.e., as evaluated by expected free energy. Regarding privacy, the agents themselves agree on what they want to share, by jointly learning the likelihood parameters of the communication modality. This can be thought of as developing their own private code, which can only be deciphered when having access to the same shared belief space. Similarly, differential privacy can be accomplished by only communicating aggregate Dirichlet counts, rather than individual observations.

This perspective on federated learning derives from research on the emergence and dynamics of human communication in computational neuroscience and evolutionary cognitive anthropology. The ensuing approach may enable the design of generic agents that draw their data from different, complementary sources, by composing a series of communicating local models into a network of belief-sharing. This helps to finesse the highly non-trivial problem of automatically designing low-cost models with the right number and configuration of parameters to enable to emergence of such a network (Friston et al., 2022b, Kauffman and Johnsen, 1991, Odling-Smee et al., 2013).

Decentralised learning—the kind of belief-sharing illustrated in our simulations—requires a high degree of similarity between agents’ beliefs, which are learned by sharing data (outcomes). This kind of individual optimization, even when based on a shared language, may not be sufficient in cases where the communication channels between agents are perturbed (e.g., communicating under water distorts the outcomes produced by agents adapted to live on land). How can one design a generic shared model, through communicative interaction, if the signals sent along agents are distorted? Thus, beyond mere similarity or even isomorphism, another condition for belief sharing is the presence of a communication channel that itself is able to maintain the integrity of observable outcomes in the environment; namely, a shared, likelihood model responsible for the structure of the sensory observations to be shared between agents.

This reflects the fact that communication and distributed cognition require that the *communication milieu* should be conducive to shared understanding, and speaks to a tenet of our approach. In the active inference literature, issues regarding the communication milieu are discussed under the banner of *econiche construction* (Creanza and Feldman, 2014, Krakauer et al., 2009, Laland et al., 1999, Lehmann, 2008). This highlights the symmetry of the active inference formulation, which describes not only the manner in which agents attune to their environment, but also how, on average and over time, by accumulating the traces left by actions of agents, the environment also comes to reflect the statistical structure of its denizens (Constant et al., 2018, Friston et al., 2022b, Veissiere et al., 2019). We pursue this from the point of view of ethology.

### 6.4 Cultural niche construction: a perspective from neuroethology

In evolutionary biology, econiche construction can be broadly understood as the implicit and explicit modification by organisms of their own environment (Odling-Smee et al., 2013), serving functions at multiple spatial and temporal scales. This modification comes in two flavours. At the phylogenetic scale (that of the entire species), *selective* niche construction generates new feedback loops that can steer selection pressures and change the fitness landscape in a way that benefits the agents that constructed the niche (Han and Hui, 2014). At the ontogenetic scale (of individuals and their development), *developmental* niche construction allows for the reproduction of the life cycle by securing the availability of expected developmental inputs (Stotz, 2017). At the scale of behaviour, cognitive niche construction guides the execution of cognitive functions such as perception, action and learning, by supporting the performance of those functions (Bertolotti and Magnani, 2017) and canalizing them down specific paths (Constant et al., 2018, Veissiere et al., 2019).

From the perspective of belief-sharing under active inference, at the phylogenetic level, niche construction allows for the selection of environments (i.e., a communication milieu) that best fits the communicative abilities of the agents, thereby preparing agents to synchronize to one another, albeit vicariously, through their environment over developmental time (Bruineberg et al., 2018). Over developmental and intergenerational time, explicit and implicit modifications of the econiche allow the niche to embody knowledge that can be transmitted (horizontally) to the next generation, thereby supporting synchrony and communication (Constant et al., 2018, Manrique and Walker, 2023). The general picture is that of econiche construction as a process that stabilises and maintains the communication milieu by contributing to the selection of organisms endowed with the necessary biological apparatus to sense observations characteristic of the communication milieu (i.e., the selection of the isomorphism), and also, crucially, by encoding or storing those observations in a reliable way. Cultural patterns such as written language would be cases in point of such co-evolved econiches, allowing for synchronisation and sophisticated cognitive functions such as mind reading (Heyes, 2018, Veissiere et al., 2019).

Situations—like the synthetic sentinel simulation considered above—are found in nature. Mammals and birds can exhibit referential communication, in which specific alert calls are used to communicate predator types (Gill and Bierema, 2013, Townsend and Manser, 2013). For example, Japanese great tits (*Parus minor*) use combinations of more than a dozen different notes to share the type of predator identified—including whether they are flying (e.g., crows) or approaching the nest from the ground (e.g., martens)—to broadcast the likely location of the predator (Suzuki, 2014). In the same way that humans combine a finite set of words to create compositional syntax with infinite meanings, tits also use compositional syntax, such as combining ‘scan for danger’ and ‘approach the caller’ notes (Suzuki, 2014, Suzuki et al., 2016). These observations suggest the existence of a hierarchical and factorial (neuronal) architecture that integrates shared messages to form a posterior belief. In avian vocal learning, young birds empirically learn the meaning of calls of adult birds, in which certain neural populations in the higher-level auditory cortex, called the caudomedial nidopallium, respond selectively to the learned song (Yanagihara and Yazaki-Sugiyama, 2016). This further suggests the formation of a sparse representation, consistent with a sparse likelihood mapping acquired via active learning. In short, these empirical observations imply possible targets, when testing process theories based on active inference.

### 6.5 Co-evolution: a perspective from evolutionary biology

In the simulations above, communication among agents was auditory and synchronous. Federated learning can also occur though other sensory modalities, and importantly through the asynchronous engagement of multiple agents within a jointly-modified niche. For example, in the case of an ant colony, the distribution of pheromones around the nest can be considered as an extended colony-level memory system, akin to an inscribed symbolic language. In this stigmergic setting, the learning process occurs among nestmates indirectly, through their ongoing contact with the niche. From the perspective of an ant nestmate’s generative model, the observed pheromone distribution provides valuable information that guides their next movements (Friedman et al., 2021), reflecting learned or inherited associations between that pheromone and its (semantic) meaning. From the perspective of the environment, the modified niche can be seen as a trace of the statistical structure of the ant colony, in line with the agent-niche symmetry discussed above: c.f., (Bruineberg et al., 2018). This type of extended and federated cognition underlies the distributed physiology found in eusocial insect colonies, enabling collective intelligences with scale and scope far beyond any nestmates body (Friedman et al., 2020).

When considering all the resources of active inference, one can see how federated systems—implementing belief-sharing and econiche construction—may allow efficient training: individual agents learning their respective local model based on shared observations, which would be encoded themselves through a learning process in a generative process that would function as the econiche. Intergenerational transmission of developmental tendencies considered as Bayesian model selection, neatly connects the computational challenges and affordances of federated learning with modern perspectives in evolutionary biology (Friston et al., 2023b).

### 6.6 Collective intelligence and group cognition: a perspective from complex dynamical systems

This work also sits in close relation to the study of collective intelligence and collective computation in natural and artificial systems. Many popular models of collective inference and learning cast agents as driven by simple rules for transforming environmental and social information into individual decisions, which translate to collective outcomes (Beckers et al., 1990, Couzin et al., 2005, Pratt and Sumpter, 2006, Strandburg-Peshkin et al., 2015). Under the formalism adopted here, these decision rules might be re-cast as approximations to a form of belief-sharing among agents about some shared latent states of the environment (Albarracin et al., 2022, Heins et al., 2023, Krafft et al., 2021).

In contrast to the explicit belief-sharing approach adopted for the current simulations, in many natural collective scenarios (e.g., in animals without explicit communication modalities), these common signals may only indirectly relate to shared contextual variables, and often manifest as noisy, ambiguous sensory input to conspecifics (Couzin et al., 2005, Pérez-Escudero and de Polavieja, 2011, Torney et al., 2015). This sort of indirect information sharing has consequences for group intelligence and performance; for example, by introducing the risk of amplifying noisy or irrelevant information (Albarracin et al., 2022, Couzin et al., 2011, Poel et al., 2022, Sosna et al., 2019). In the context of multi-agent active inference, such maladaptive group outcomes may correspond to local minima in a joint free energy landscape—while individuals may sit at fixed points in their (private) free energy functionals, these fixed points may not always coincide with fixed points in the group’s free energy landscape (Heins et al., 2023).

Further research might help identify the conditions on what types of sensory information channels (and corresponding likelihood models) are sufficient to enable the group to perform optimally, e.g., in the context of consensus decision making or collective perception (Berdahl et al., 2013, Hein et al., 2015, Ward et al., 2008). Furthermore, how exactly conspecifics should update their generative models (e.g., through parameter learning or structure learning) to minimize collective free energy, as opposed to individual-level free energy, is an open question.

### 6.7 How the desire to share creates language: a perspective from psychology

Most of our beliefs about the world come, not from direct experience, but from other people. All animals, from fruit flies to humans, learn about the world by observing others (Frith, 2010, Kilner et al., 2007, Manrique and Walker, 2023, Rieucau and Giraldeau, 2011). For example, they learn where to go and what to eat. The effect of this learning is that the children will come to share the priors of their parents and of their culture more generally. But humans are unique, since we can learn about the world from others through verbal instruction. Such instruction depends upon communication, mostly via language.

Humans are incredibly social animals (Manrique and Walker, 2023, Torney et al., 2015, Vasil et al., 2020). We not only want to be *liked* by our friends and neighbours (our in-group). We want to be *like* them. And the best way to be like others is to align with them. We achieve such alignment at many levels. At the physical level, we dance, negotiate the city streets, and move furniture together. At a more abstract level, we share our goals. And at the highest, mental level we share our ideas and, in particular, our models of the world. Many advantages accrue from sharing models of the world. Because of our complementary perspectives, our shared models will be richer and more accurate than any individual model. Furthermore, having shared models, and common ground more generally, communication becomes easier (Bahrami et al., 2010, Clark and Brennan, 1991, Frith and Wentzer, 2013, Heyes and Frith, 2014).

In this context a virtuous circle is created. The desire to share encourages the emergence of language. Language enlarges our common ground and makes our models of the world more accurate. This creates a mental niche in which communication is more efficient and our ability to share is enhanced. And all this can be achieved—or, strictly speaking, described—by free energy minimisation.

### 6.8 Constraints and communication: a perspective from linguistics

The level of analysis—in this treatment of communication—falls short of addressing linguistics per se. However, there are some crosscutting themes in classical linguistic theories such as Universal Grammar (Chomsky, 2017) and Optimality Theory (Prince and Smolensky, 2007) that deserve mention. Universal Grammar rests on the notion of innate *constraints* on the grammar of possible languages. Similarly, Optimality Theory (Prince and Smolensky, 2007) suggests that the observed forms of language arise from the optimal satisfaction of *constraints*. Both approaches foreground the role of constraints that—from the perspective of communication under active inference—arise naturally from free energy minimisation. For example, the free energy principle is dual to the *constrained* maximum entropy principle (Sakthivadivel, 2022a), where the constraints inherit from the generative model. Intuitively, if language is the broadcasting of beliefs under a generative model, then the structure of language should inherit the factorial (and deep) structure of the underlying generative model. In turn, the structure is constrained by the causal structure of the lived world within which we act—a world that includes our bodies and conspecifics.

On this view, it is literally self-evident (in the sense of self-evidencing) that language is subject to innate constraints; constraints that inherit from learning the structure of generative models apt to explain the co-constructed world. These models necessarily have a grammar; in the sense of dynamics and non-Markovian temporal structure, as evinced in hierarchical structure learning treatments. See for example: (Davis and Johnsrude, 2003, Friston et al., 2017c, George and Hawkins, 2009, MacKay and Peto, 2008, Stoianov et al., 2022, Yildiz et al., 2013, Young et al., 2018, Zorzi et al., 2013). In short, grammar and meaning should be isomorphic with world models.

This view also speaks to the Symbol Grounding Problem (Harnad, 1990); namely, how words acquire meaning. The problem of meaning is dissolved if one commits to the notion that words are (overt or covert) declarations of beliefs, and beliefs are over discrete states. In other words, the meaning of a word just is [isomorphic to] the beliefs about the current state of the world and the narratives inherent in state transitions. The symbol grounding problem is dissolved in the sense that words are both cause and consequence of world models that underwrite an active engagement with the world. The emergence of shared meaning (c.f., common ground) then reduces to an alignment of the (likelihood) mapping from belief states to words or symbols, among correspondents. It is interesting to speculate about the impact of generative artificial intelligence (AI) in general, and large language models in particular, in light of this view. For example, are the implicit generative models in generative AI sufficiently expressive to constitute a world model that includes the consequences of their action? E.g., (Chalmers, 2023).

## 7 Conclusion

The account given above offers several key advances on state-of-the-art active inference and structure learning. The technical contributions of this paper are twofold. The first is belief-sharing—the idea that biological or artificial agents with different vantage points can communicate their inferences and inform one another about their shared environment. We saw this in the context of agents, with different viewpoints, speaking to one another to share their percepts to better characterise their world. The second axis is an intelligent procedure for updating beliefs, at the level of learning and model selection. This procedure—based upon the prior belief that our world should be precise, predictable, and sparse— furnishes a (common) sense of purpose in both learning and selection. When combined, these two advances offer a powerful form of federated belief-optimisation that may shed light on biological development and the design of intelligent (eco) systems in general, and belief sharing in particular.

## Software note

Although the generative model – specified by the (A,B,C,D) matrices – changes from application to application, the belief updates in Fig. 2 are generic and can be implemented using standard routines (here **spm_MDP_VB_XXX.m**). These routines are available as Matlab code in the SPM academic software: http://www.fil.ion.ucl.ac.uk/spm/, or available from https://github.com/spm/.

## Footnotes

1 For clarity, we will use the pronoun ‘she’ for agents and ‘it’ for things (e.g., subjects) that agents have beliefs about.

2 To avoid disrupting the narrative, we will put technical details—concerning variational message passing and generative models—in figure legends. This allows us to focus on the conceptual foundations and key functional forms of requisite (free energy minimising) processes.

3 Note that in this setting, a policy is not a sequence of actions but simply a combination of paths, where each hidden factor has an associated state and path. This means there are, potentially, as many policies as there are combinations of paths.

4 The ⊙ notation implies a sum product operator; i.e., the dot or inner product that sums over one dimension of a numeric array or tensor. In this paper, these sum product operators are applied to a vector **a** and a tensor **A** where, a⊙A implies the sum of products is taken over the leading dimension, while A⊙aimplies the sum is taken over a trailing dimension. For example, 1⊙Ais the sum over columns and A⊙1is the sum over rows, where 1is a vector of ones. This notation replaces the Einstein summation notation to avoid visual clutter.

5 For deterministic outcomes one can read Q(o,a)=δ(o)Q(a), where the Kronecker delta function, δ(o) is a one-hot vector encoding the observation in question. Otherwise, for outcomes that are provided probabilistically, we have Q(o,a)=P(o)Q(a):P(o)=Cat(o).

6 In other words, instead of supplying observations as a one-hot vector, auditory observations are supplied as probability distributions over the discrete outcomes of each modality; i.e., the sufficient statistics of categorical distributions.

7 For simplicity, we assume tensors have been formatted as matrices, by vectorising the second and subsequent dimensions.

8 Expected free energy gradients are taken with respect to log Dirichlet counts because Dirichlet concentration parameters are nonnegative scale parameters.

9 Roughly speaking, a variational free energy of 3 would be considered surprising in the sense of a Bayes factor, relative to perfect predictions Kass, R.E., Raftery, A.E., 1995. Bayes Factors. Journal of the American Statistical Association 90, 773–795.

10 Extensive in the sense that the total free energy is the sum of local free energies.

11 Where complexity is read as the relative entropy or KL divergence between posterior and prior beliefs.

12 The free energy due to learning in Fig. 7 scores the increase in complexity after each episode.
